# Linguistic information may compensate for age-related decline in attentional filtering

**DOI:** 10.1038/s42003-026-10999-y

**Published:** 2026-09-22

**Authors:** Alice Vivien Barchet, Andrea Bruera, Johanna M. Rimmele, Jonas Obleser, Gesa Hartwigsen

**Affiliations:** 1 Research Group Cognition and Plasticity, Max Planck Institute for Human Cognitive and Brain Sciences, Leipzig, Germany; 2 Cognitive and Biological Psychology, Leipzig University, Leipzig, Germany; 3 International Max Planck Research School on Cognitive NeuroImaging (IMPRS CoNI), Leipzig, Germany; 4 Department of Cognitive Neuropsychology, Max Planck Institute for Empirical Aesthetics, Frankfurt, Germany; 5 Department of Psychology, University of Luebeck, Luebeck, Germany; 6 Center of Brain, Behavior, and Metabolism, University of Luebeck, Luebeck, Germany

**Keywords:** Attention, Cognitive ageing, Language

## Abstract

As we age, understanding speech in social situations imposes an increasingly difficult challenge to the auditory system. However, the attentional mechanisms underlying age-related speech comprehension difficulties in multitalker situations remain unclear. We collected EEG signals while 63 normal hearing participants (35 women) from 19 to 71 years performed a speech comprehension task involving a multitalker paradigm at individually adjusted target-to-distractor ratios. Combining trial-resolved multivariate temporal response function modeling with detailed behavioral comprehension responses, we provide a window into lower-level impairments and possible higher-level compensatory mechanisms across the adult life span. We show that reduced behavioral performance in late adulthood is associated with increased distractor representation. This points towards increased distractibility as a potential mechanism underlying age-related speech comprehension deficits. Additionally, at the behavioral and neural levels, we show that older adults rely more on higher-level linguistic information. Finally, we show that an increased reliance on word-level linguistic information may compensate for increased distractor tracking and adaptively support comprehension performance across the adult life span. Thus, an increased reliance on higher-level processing could attenuate age-related impairments in attentional filtering.

## Introduction

In everyday life, spoken speech is often masked by noise or competing speech streams in the surrounding environment, creating challenging listening situations, particularly for older adults^1–3^. However, it remains unclear why older adults’ speech comprehension is impaired in multitalker contexts. Disentangling the causes of impaired speech comprehension is particularly important as impaired hearing abilities are associated with poor cognitive and psychosocial functioning in late adulthood^4,5^.

Healthy aging results in many changes in sensory processing, including hearing loss and reduced auditory temporal resolution^6–10^. At the same time, speech comprehension remains largely preserved across the adult lifespan^11,12^. However, older adults often experience difficulties in perceptually demanding listening conditions, such as competing speech situations^11–13^. Successful speech comprehension in challenging listening situations requires separating the to-be-attended target stream from one or more to-be-ignored distractor streams. Speech comprehension seems to be related to low-frequency cortical activity that temporally aligns with the speech signals^14–16^. In competing speech comprehension, neural tracking of acoustic properties of the to-be-attended signal has been shown to be selectively enhanced^17–21^. Recent evidence suggests that this attentional filtering is particularly susceptible to senescent changes, potentially due to impaired neural inhibition ^22,23^.

Behavioral studies have suggested that older adults rely more on contextual cues than younger adults to maintain speech comprehension ability despite peripheral declines^13,24,25^. Correspondingly, processes involved in the activation of contextual knowledge have been shown to be relatively unaffected by aging^26,27^. Yet, neural results employing event-related potentials have revealed weaker responses to semantic context in older adults than in younger adults^28–30^. Interestingly, recent methodological advances have enabled researchers to investigate neural speech tracking in natural speech on several hierarchical levels, including acoustic, phonemic, and semantic information in a unified encoding framework^31–34^. Here, neural results concerning the representation of semantic information across the adult lifespan have revealed heterogeneous results, suggesting that semantic information might be represented more strongly^35^, similarly^36^, or less strongly^37^ in older adults’ brain signals. In contrast, the neural tracking of acoustic properties is typically observed to be stronger in older adults^37–40^. However, it is unclear whether this increased tracking of acoustic properties serves a compensatory purpose or if it reflects increased processing noise^22,38,41–43^.

To disentangle neural mechanisms of age-related speech comprehension deficits from compensatory mechanisms, it is crucial to link measures of neural tracking with behavioral comprehension performance. However, such a link is missing in most previous studies. Most studies investigating speech tracking in naturalistic contexts lack detailed behavioral responses and are therefore unable to relate neural tracking to time-resolved variations in comprehension success^34–37,39^. Here, we combined time-resolved behavioral outcome measures and EEG using a naturalistic competing speech paradigm to probe the behavioral relevance of acoustic and higher-level processing across the adult lifespan. In contrast to previous studies that used natural listening paradigms, we asked participants to listen to short sentences and repeat the words they understood. This allowed the analysis of comprehension dynamics using a fine-grained, word-by-word intelligibility measure. Additionally, task difficulty was individually adjusted based on the individual speech reception threshold (SRT) as measured by an adaptive staircase procedure to control for effects of peripheral constraints. As a result, the average comprehension performance was balanced across participants to allow for conclusions regarding the use of different strategies to achieve similar performance levels. Using multivariate temporal response function (TRF) modeling, we provide insight into the trial-wise neural tracking strength of acoustic and linguistic speech representations for the target and distractor streams, as well as their relationship to behavioral comprehension performance and changes of this relationship across the adult lifespan.

By employing trial-resolved analyses on the behavioral and neural levels, we addressed the overarching research question of the mechanisms underlying age-related changes in speech comprehension in multitalker environments. Investigating speech features at different hierarchically organized levels allowed us to disentangle how lower-level and higher-level linguistic mechanisms support comprehension performance in a challenging and naturalistic multitalker paradigm. Here, lower-level mechanisms refer to phonetic (i.e., envelope) and phonological (i.e., word and phoneme onsets) processing. Higher-level mechanisms refer to responses to probabilistic features, such as word frequency, word surprisal, and word entropy.

Based on previous evidence suggesting age-related changes in temporal resolution and hearing acuity, we expected older adults to display lower-level impairments potentially affecting their attentional filtering strategies. Additionally, we hypothesized that in a multitalker environment like the one we tested here, older adults would engage in a compensatory recruitment of higher-level mechanisms in speech comprehension. Specifically, older adults should demonstrate a stronger reliance on higher-level information on the behavioral and the neural levels.

Our results demonstrate that the neural tracking of the distractor stream may serve as a behaviorally relevant indicator of age-related impairments in stream segregation. At the same time, behavioral and neural results indicate a stronger reliance on higher-level target processing with increasing age. Collectively, our results highlight the interplay of lower-level impairments and higher-level compensation across the adult lifespan.

## Results

### Behavioral results

#### Both lower-level and higher-level features predict comprehension performance

**Fig. 1:**
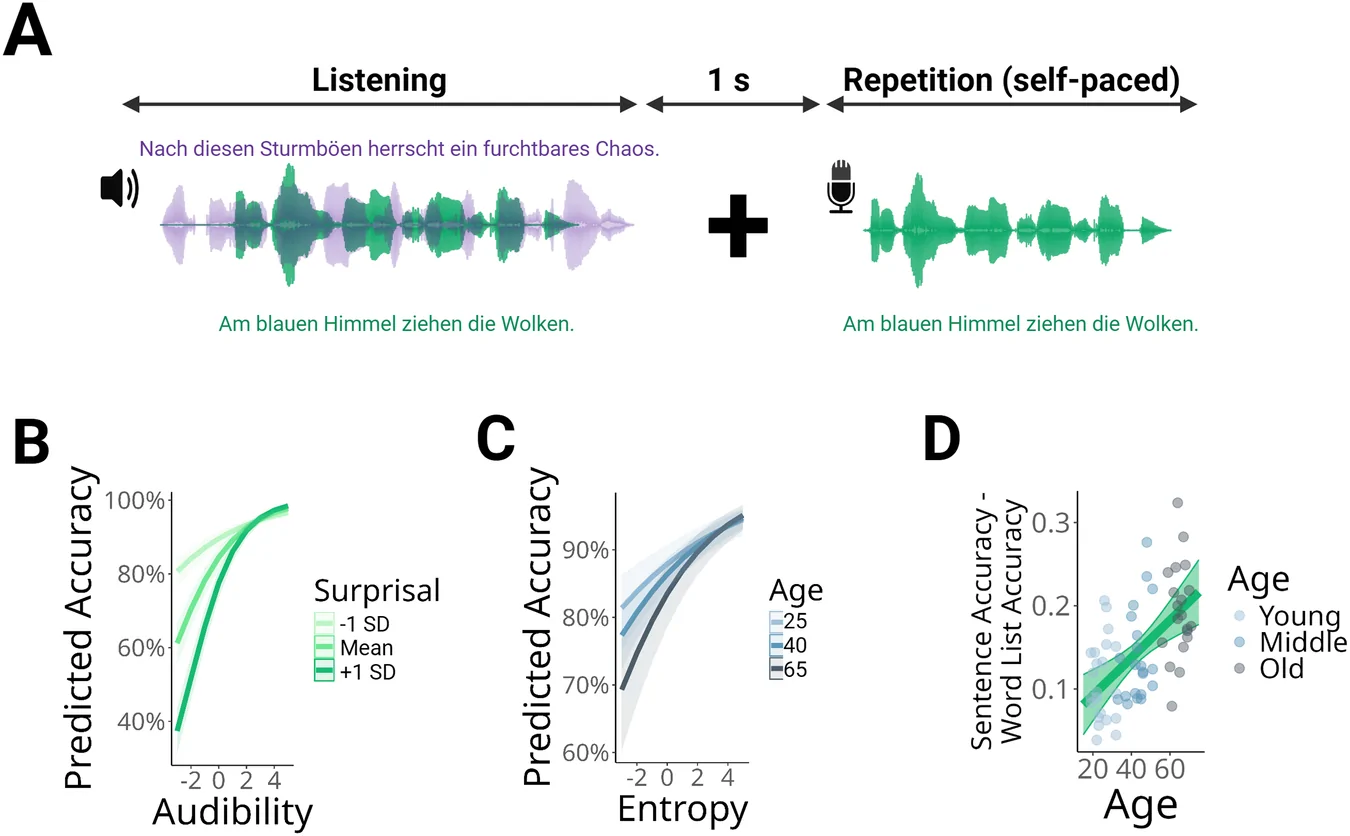
Behavioral analysis. **A** Behavioral Paradigm. Participants (*N* = 63) listened to 240 trials comprised of one target and one distractor sentence. They subsequently repeated the target sentence. English translations of the sentences: After these squalls, there is a terrible chaos (purple), Clouds gather in the blue sky (green). **B** Effects of surprisal and audibility on comprehension accuracy. Surprisal and audibility jointly predict comprehension accuracy, with a bigger effect of surprisal at low levels of audibility (Estimate = 0.17, SE = 0.02, *p* < 0.001**). The effect was modeled using a generalized linear mixed-effects model at the word level, predicting word-by-word comprehension accuracy. Statistical significance of fixed effects was assessed using two-sided Wald tests. *P*-values were corrected for multiple comparisons using FDR. **C** Interaction between age and word entropy. Word entropy had a stronger positive effect on comprehension accuracy in older adults than in younger adults (Estimate = 0.04, SE = 0.02, *p* = 0.008**). The effect was modeled using a generalized linear mixed-effects model at the word level, predicting word-by-word comprehension accuracy. Statistical significance of fixed effects was assessed using two-sided Wald tests. *P*-values were corrected for multiple comparisons using FDR. Data are presented as the estimated marginal effects ± 95% confidence intervals. **D** Comprehension gain from sentence context and age. The accuracy difference between the main experiment and an additional block of word lists was larger with increasing age (Estimate = 0.60, SE = 0.16, *p* < 0.001**). The effect was investigated in a linear mixed-effects model at the subject level, predicting the standardized accuracy difference from age. Age was used as a continuous predictor in all analyses, and age groups were used for illustrative purposes only. *P*-values were corrected for multiple comparisons using FDR. Data are presented as the estimated marginal effects ±  95% confidence intervals. Source data are provided in Supplementary Data 1.

In a generalized linear mixed model, we predicted word-by-word comprehension performance from word-level audibility, surprisal, and entropy for the target stream. Results revealed that audibility, surprisal, as well as their interaction, predicted word repetition performance. Figure 1B displays the interaction between word audibility and surprisal, indicating that the negative effect of surprisal is stronger at low levels of audibility, with higher comprehension for low surprisal (i.e., more predictable) words. Word entropy positively predicted comprehension performance. While this effect seems counterintuitive, it is consistent with previous studies showing more efficient lexical processing in contexts of high uncertainty^44,45^. This effect could indicate that the allocation of attention as well as the pre-activation of semantic features is increased in contexts of high uncertainty. Overall, there were negative effects of age and pure tone average (PTA) on comprehension performance. Complete results for the generalized linear mixed model can be retrieved from Table 1.

**Table 1:** Model outputs predicting comprehension performance

	Estimate	Std. error	CI	*z*	*p*
**Behavioral effects**					
Age	−0.55	0.08	[−0.71, −0.39]	−6.70	<0.001
Audibility	0.41	0.01	[0.38, 0.44]	27.82	<0.001**
Surprisal	−0.46	0.02	[−0.5, −0.42]	−22.31	<0.001**
Audibility:Surprisal	0.17	0.02	[0.14, 0.2]	11.09	<0.001**
Word frequency	0.15	0.02	[0.1, 0.19]	6.02	<0.001**
Word entropy	0.21	0.02	[0.17, 0.24]	10.97	<0.001**
Audibility:Age	0	0.01	[−0.03, 0.02]	−0.38	0.761
Surprisal:Age	0	0.02	[−0.03, 0.03]	0.17	0.868
Entropy:Age	0.04	0.02	[0.01, 0.07]	2.80	0.008**
**Lower-level TRF fits**					
Acoustic target	0.02	0.01	[−0.01, 0.04]	1.49	0.172
Acoustic distractor	0.03	0.01	[0.01, 0.05]	2.29	0.032*
Onsets target	0.05	0.01	[0.03, 0.07]	4.29	<0.001**
Onsets distractor	−0.02	0.01	[−0.05, 0.00]	−2.11	0.049*
Acoustic target:Age	0.01	0.01	[−0.01, 0.03]	0.74	0.533
Acoustic distractor:Age	0.00	0.01	[−0.02, 0.03]	0.32	0.776
Onsets target:Age	−0.02	0.01	[−0.05, 0.00]	−1.99	0.062
Onsets distractor:Age	−0.03	0.01	[−0.05, −0.01]	−2.48	0.021*
**Higher-level TRF fits**					
Word target	0.03	0.01	[0.01, 0.06]	2.87	0.007**
Word target:Age	0.01	0.01	[−0.02, 0.03]	0.62	0.596
**Control variables**					
Pure tone average	−0.36	0.08	[−0.52, −0.21]	−4.68	<0.001**
Target-to-distractor ratio	0.49	0.01	[0.47, 0.51]	55.86	<0.001**
Trial order	0.09	0.01	[0.07, 0.12]	8.08	<0.001**
Word length	0.23	0.02	[0.19, 0.27]	11.65	<0.001**
Word order	−0.24	0.02	[−0.27, −0.20]	−13.37	<0.001**
Sentence length	−0.08	0.08	[−0.24, 0.09]	−0.90	0.448
Control accuracy	0.25	0.07	[0.11, 0.39]	3.43	0.001**
Speech reception threshold	0.83	0.09	[0.66, 1.00]	9.49	<0.001**

Results from one logistic mixed-effects model predicting the behavioral comprehension accuracy from speech features, trial-wise neural tracking estimates, and control variables (*N* = 63 participants).

**p < 0.01, *p < 0.05 (FDR corrected).

#### Stronger behavioral reliance on entropy in older adults

Additionally, the model revealed an interaction between age and word entropy. This interaction indicates that the effect of word entropy on comprehension was stronger with increasing age (Fig. 1C). More precisely, there seems to be a stronger negative effect of low word entropy on comprehension performance with increasing age. This effect suggests that older adults’ comprehension performance is modulated more strongly by the linguistic context.

#### Comprehension gain from sentence context increases with age

To quantify how much each participant’s behavioral accuracy benefited from sentence context, we calculated standardized differences between the mean accuracy in the main experiment and an additional block of lists of unrelated words. A multiple linear regression model predicting these difference scores from age revealed a positive correlation between age and the accuracy gained from sentence context (Estimate = 0.60, SE = 0.16, *t* = 3.85, *p* < 0.001). The effect is visualized in Fig. 1D. Thus, in a noisy environment, older adults seem to benefit more than younger adults from the richer linguistic context provided by meaningful, coherent sentences as opposed to unrelated sets of words.

### Lower-level TRF model fits

#### Significant lower-level tracking for the target and distractor streams

**Fig. 2:**
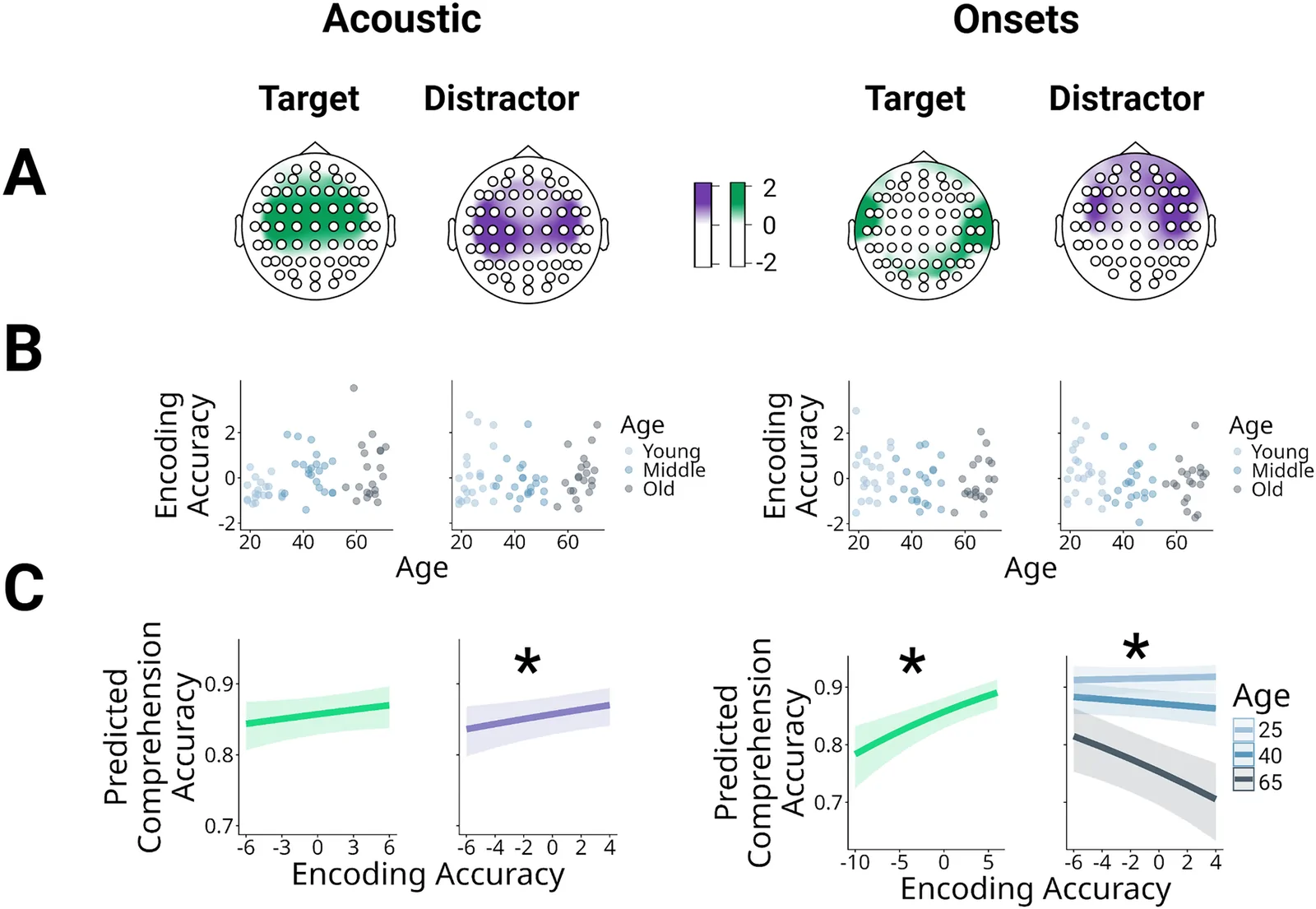
Neural tracking and neuro-behavioral correlations for the lower-level features. **A** Topographies for the encoding accuracies. Significant electrodes in one-sided cluster-based permutation *t*-tests are marked by white dots. Statistical testing was performed at the participant level (*N* = 63 participants). **B** Age effects on encoding accuracy. No significant main effects of age on encoding accuracy strength were observed. Effects were investigated using separate linear mixed-effects models at the subject level predicting model fit from age. **C** Neuro-behavioral correlations and age interactions. Generalized linear mixed model predictions of the effects of trial-wise neural encoding accuracy on word-by-word comprehension accuracy. Distractor acoustic features (Estimate = 0.03, SE = 0.01, *p* = 0.032*) and target onsets (Estimate = 0.05, SE = 0.01, *p* = < 0.001**) showed a positive relationship with comprehension accuracy. A significant interaction between the encoding model fits and age emerged for the distractor onsets (Estimate = −0.03, SE = 0.01, *p* = 0.021*), suggesting that older adults' comprehension performance is more negatively influenced by the neural tracking of distractor features. Statistical significance of fixed effects was assessed using two-sided Wald tests. *P*-values were corrected for multiple comparisons using FDR. Data are presented as the estimated marginal effects ± 95% confidence intervals. Source data are provided in Supplementary Data 1.

To investigate the neural tracking of lower-level features, we conducted a TRF analysis predicting the EEG signals from acoustic features and word and phoneme onsets. Acoustic features and onsets were shuffled separately to disentangle acoustic processing and word and phoneme segmentation. As displayed in Fig. 2A, the lower-level features were significantly tracked for the target and distractor streams. Accordingly, significant encoding model performance with increased model fit compared to a shuffled null distribution was observed for the acoustic features and the word and phoneme onsets. The topographies reveal the strongest encoding accuracies in frontal and central sensors.

#### No effects of age on lower-level encoding accuracy

To assess the effects of age on encoding accuracy, we used the mean encoding accuracy of the lower-level models across all sensors. We used multiple linear regression models predicting the mean encoding accuracy for every participant from age. The analysis was controlled for the participant-specific SRT, the accuracy in the main experiment and in the control condition, and hearing ability as measured by the PTA. The control condition consisted of well-intelligible sentences presented at SRT +10 dB. There were no significant associations between age and the strength of lower-level encoding accuracies.

#### Stronger lower-level encoding is related to better comprehension

Using a generalized linear mixed model, we predicted word-by-word comprehension accuracy from the trial-wise TRF model fits, as well as their interactions with age. We observed positive effects of lower-level tracking on comprehension performance for the target and distractor streams. More precisely, the neural tracking of target word and phoneme onsets and the tracking of distractor acoustics were positively related to comprehension accuracy. The model predictions are visualized in Fig. 2C. First, these effects show that word and phoneme segmentation of the target stream is crucial for comprehension. Second, increased acoustic tracking of the distractor stream was related to improved comprehension performance across the full age range. This could indicate that an initial acoustic analysis of the distractor stream and segmentation of the target stream are beneficial for stream segregation and comprehension performance.

#### Increased age correlates with stronger distraction

The generalized linear mixed model revealed a significant interaction between the effects of age and the neural tracking of word and phoneme onsets in the distractor stream on comprehension performance. A post-hoc simple slopes analysis revealed a significant negative effect of the neural tracking of distractor onsets in older adults (slope of distractor onsets when age = 65: Estimate = −0.06, SE = 0.02, *z* = −3.28, *p* = < 0.01**), but not in younger (slope of distractor onsets when age = 25: Estimate = 0.01, SE = 0.02, *z* = 0.40, *p* = 0.69) or middle aged adults (slope when age = 40: Estimate = −0.02, SE = 0.01, *z* = −1.52, *p* = 0.13).

Thus, a stronger neural representation of distractor onsets had a negative effect in older adults, but not in younger adults. At this intermediate level of (sub-)lexical segmentation, older adults’ comprehension performance seems to be negatively affected by the neural tracking of the distractor sentence. This indicates that an increased allocation of attention to the distractor stream occurring at an intermediate level might underlie older adults’ speech comprehension difficulties. The effect is visualized in Fig. 2C. Although the tracking of distractor acoustics was positively related to comprehension, we observed a negative effect of distractor onsets in older adults. This might indicate that, while acoustic analysis is required for comprehension, tracking of the intermediate onset level marks a distraction effect that is detrimental for comprehension.

### Higher-level TRF model fits

#### Word-level features are significantly tracked for the target stream

**Fig. 3:**
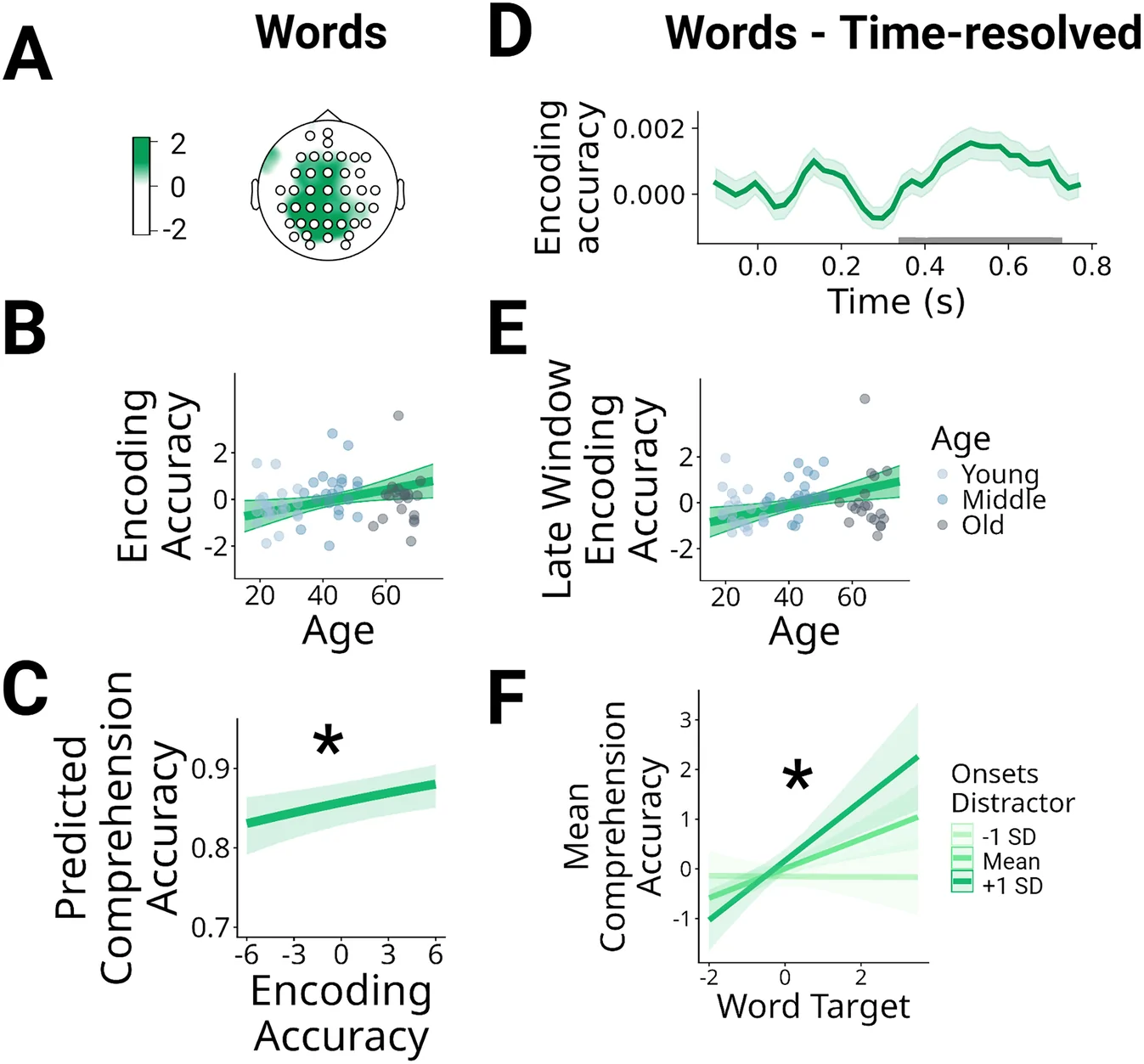
Neural tracking and neuro-behavioral correlations for the higher-level target features. **A** Topographies for the encoding accuracies. Significant electrodes in one-sided cluster-based permutation *t*-tests are marked by white dots. Statistical testing was performed at the participant level (*N* = 63 participants). **B** Stronger higher-level linguistic tracking with increasing age. There was a positive correlation between age and word-level encoding accuracy (Estimate = 0.43, SE = 0.19, *p* = 0.024*). The effect was investigated using a linear mixed-effects model at the subject level, predicting model fit from age. **C** Neuro-behavioral correlations. Generalized linear mixed model predictions of the effects of trial-wise neural encoding accuracy on word-by-word comprehension accuracy revealed a positive effect of word-level target tracking on comprehension accuracy (Estimate = 0.03, SE = 0.01, *p*_FDRcorrected_ = 0.007**). Statistical significance of fixed effects was assessed using two-sided Wald tests. Data are presented as the estimated marginal effects ± 95% confidence intervals. **D** Time-resolved encoding accuracy for the target stream and age effect on encoding accuracy in a late window (400–800 ms). Gray bars indicate significant encoding performance resulting from a one-sided cluster-based permutation one-sample *t*-test with threshold-free cluster enhancement. Family-wise error rate correction for multiple comparisons was achieved through the permutation-based cluster procedure. Data are presented as the mean ± standard errors around the mean. **E** Stronger late word-level tracking with increasing age. There was a positive correlation between age and word-level encoding accuracy in a late encoding window starting at 400 ms after word onset (Estimate = 0.51, SE = 0.18, *p* = 0.006**). The effect was investigated using a linear mixed-effects model at the subject level, predicting model fit from age. Data are presented as the estimated marginal effects ± 95% confidence intervals. **F** Word-level tracking may compensate for increased distraction. Significant interaction between the tracking of word-level target features and distractor onsets (Estimate = 0.30, SE = 0.09, *t* = 3.27, *p*_FDRcorrected_ = 0.006**) in a linear mixed-effects model at the subject level predicting subject comprehension accuracy. The positive effect of word-level target tracking on comprehension accuracy was larger at high levels of distractor-onset tracking, indicating a potential compensatory function of higher-level tracking. Statistical significance of fixed effects was assessed using two-sided *t*-tests with Satterthwaite approximations for degrees of freedom. Data are presented as the estimated marginal effects ± 95% confidence intervals. Source data are provided in Supplementary Data 1.

To investigate the neural tracking of higher-level features, we conducted a separate TRF analysis predicting the residualized EEG responses from word-level (word frequency, surprisal, and entropy) and phoneme-level (phoneme surprisal and phoneme entropy) probabilistic features. As displayed in Fig. 3A, the word-level probabilistic features were significantly tracked for the target stream. This was expected based on previous results and indicates a successful allocation of attention towards the target stream, at least in the majority of trials (see ref. ^46^ for an exploration of the neural tracking of the distractor stream resolved by comprehension accuracy). The phoneme-level probabilistic features were not significantly tracked. Similarly, the higher-level features were not significantly tracked for the distractor stream. Therefore, we did not include the distractor higher-level tracking and the phoneme-level tracking in the subsequent analyses.

#### Stronger higher-level tracking with increasing age

As displayed in Fig. 3B, there was a positive effect of age on higher-level target tracking at the word level (Estimate = 0.43, SE = 0.19, *t* = 2.32, *p* = 0.024*). Older adults therefore seem to display stronger neural responses to higher-level linguistic features.

#### Higher-level target tracking is correlated with improved comprehension

Consistent with previous results, the linear mixed model revealed positive effects of higher-level target tracking on comprehension performance. Thus, when word-level tracking of the target stream improved, comprehension performance was improved. The model predictions are visualized in Fig. 3C. There were no age interactions with this effect, indicating that this positive relationship was stable across the adult lifespan.

#### Higher-level linguistic tracking may compensate for increased distraction

In an exploratory analysis, we tested the hypothesis that higher-level word tracking may compensate for increased neural tracking of the distractor stream. We used a multiple regression model predicting each subject’s mean accuracy from the mean encoding accuracy of the word-level target features and the tracking of the distractor onsets and acoustics and their interactions. We additionally included three-way interactions between age, word-level target encoding accuracy, and lower-level distractor encoding accuracy. The analysis was controlled for the subject-specific SRT, the PTA, and the accuracy in the control condition. Results revealed a significant interaction between the word-level target and the onset distractor encoding accuracies (Estimate = 0.30, SE = 0.09, *t* = 3.27, *p*_FDRcorrected_ = 0.006**). This interaction indicates that the positive effect of word-level target tracking on comprehension performance was stronger when the distractor-onset tracking was increased (see Fig. 3E). The effect was specific to the neural tracking of the distractor onsets, with no significant interaction emerging for distractor acoustic tracking. Thus, there seems to be a relationship between enhanced distractibility and higher-level tracking. This means that stronger higher-level tracking might compensate for increased individual distractibility. However, based on the available correlational evidence, no causal conclusions on the origin of this relationship can be drawn. There were no significant interactions between these effects and the participants’ age, which indicates that this compensatory mechanism may be a general strategy to maintain speech processing under challenging conditions across the adult lifespan. The full model outputs can be retrieved from Table S1.

#### Time-resolved encoding accuracies reveal late tracking for word-level features

To further investigate the time course of word-level feature encoding, we conducted an exploratory analysis using time-resolved encoding accuracies for the word-level model variant. This was done to confirm that the observed effects result from late linguistic responses. Results revealed that the higher-level word features were represented in late time windows starting at 400 ms after word onset. This effect was specific to the target sentence. No significant encoding performance emerged for the distractor sentence. The results are visualized in Fig. 3D.

#### Stronger late linguistic tracking with increasing age

The results revealed a positive correlation between age and the encoding accuracy of word-level target features in a late window starting at 400 ms after target word onset (Estimate = 0.51, SE = 0.18, *t* = 2.86, *p* = 0.006**). These late time windows have been commonly associated with higher-level processes related to word identity and the processing of word expectations. Thus, the neural tracking of higher-level processes seems to increase across the adult lifespan, suggesting a stronger reliance on higher-level processing with increasing age.

## Discussion

By combining detailed behavioral comprehension responses and multivariate, trial-resolved neural encoding models, we investigated age-related changes in the behavioral and neural mechanisms underlying speech comprehension in multitalker environments. The results revealed two main findings: on the one hand, older adults showed signs of impaired stream segregation abilities and enhanced susceptibility to distraction. This behaviorally relevant impairment likely contributes to age-related comprehension difficulties in multitalker environments. On the other hand, we found stronger behavioral and neural engagement with higher-level linguistic information with increasing age. Connecting these findings, we revealed evidence that higher-level tracking may compensate for stronger neural distractibility by facilitating successful comprehension.

Collectively, our results reveal a complex pattern of age-related changes in lower-level mechanisms and increased higher-level recruitment. Increased higher-level recruitment may serve a compensatory function for an individual’s increased susceptibility to distraction. These results thus provide detailed insights into age-related impairments and potential compensatory strategies which help to maintain efficient speech processing across the adult lifespan.

Behavioral results show that comprehension performance was influenced by lower-level word audibility, as well as word surprisal and word entropy. In addition, there was an interaction between word audibility and word surprisal indicating that the effect of surprisal was stronger at lower levels of audibility. The magnitude of this behavioral effect remained constant across the adult lifespan. Thus, on a word-by-word level, older adults were equally able to use acoustic and linguistic information in order to support speech comprehension.

Additionally, there was a positive effect of word entropy on comprehension performance, indicating that comprehension improved when uncertainty was higher. This effect got stronger with increasing age, which might indicate that dynamic modulation of attention in relation to uncertainty increases with age. This means that the allocation of attention in response to highly uncertain contexts, as well as the deallocation of attention in contexts deemed rather certain, seems to be stronger in older adults. Thus, the context certainty seems to be a stronger predictor of older adults’ comprehension performance. In line with this conclusion, older adults seemed to benefit more from overall sentence context when comparing comprehension performance in sentences and word lists. These results concur with behavioral effects suggesting a stronger reliance on contextual cues in older adults^13,24,25^.

We investigated neural tracking for the target and distractor streams across several levels of the speech hierarchy, from phonetic and phonological (lower-level) mechanisms to higher-level probabilistic features. Neuro-behavioral correlations revealed that the trial-by-trial neural tracking of target word and phoneme onsets was positively associated with comprehension performance, consistent with previous results^47–52^. Additionally, we observed a positive effect of the neural tracking of distractor acoustics on comprehension. This effect suggests that an initial lower-level analysis of the distractor stream is beneficial for stream segregation. For a detailed discussion of this effect, see ref. ^46^.

Increased tracking of higher-level word features for the target stream was correlated with better comprehension performance. This is consistent with previous results^32,48,53–55^ and indicates that, as expected, when participants understood the sentences, this was reflected in a stronger neural tracking of the corresponding word-level features.

Our results did not reveal any interactions between age and the effects of acoustic or word-level encoding on comprehension, indicating that the effects of the encoding strength on comprehension performance remain constant across the adult lifespan. The reliance on acoustic and higher-level linguistic cues to parse the speech signal as the basis for speech comprehension thus seems to be stable across the adult lifespan.

Using neuro-behavioral correlations on a trial-by-trial level, we showed that the neural tracking of distractor word and phoneme onsets had a negative effect on behavior in older adults but not in younger adults. Thus, poorer speech comprehension in older adults could be explicitly associated with impaired attentional filtering and decreased neural inhibition of the distractor stream^22^. Similar findings have been observed for hearing-impaired populations^23,56,57^, suggesting that reduced stream segregation could be related to peripheral declines. Importantly, unlike previous studies^22,38,56^, we provide a missing link between the neural tracking of the distractor stream and the behavioral comprehension performance. By employing neuro-behavioral correlations on a trial-by-trial level, we show that the enhanced tracking of distractor onsets is detrimental to comprehension, particularly in late adulthood. This effect emerged while task difficulty was individually adjusted on the basis of each individual’s SRT. Thus, differences in neural distractibility cannot be attributed to an overall poorer performance in older adults due to peripheral hearing constraints. This result further reinforces evidence indicating that impaired speech comprehension in older adults might rely on impaired attentional filtering^22,24,58^. This could be related to age-related declines in the ability to inhibit the neural processing of task-irrelevant information^22,38^.

A positive correlation between age and the strength of word-level encoding for the target stream indicates that the neural tracking of higher-level probabilistic features was stronger in older adults than in younger adults. Using a time-resolved measure of encoding accuracy, we found that, when word-level probabilistic features could reliably predict brain activity (in a late processing window starting at 400 ms after word onset), a positive association between age and word-level encoding performance emerged. This late processing window is typically associated with the processing of expectations and word identity^30,48,59–61^. Combined with our behavioral findings of an enhanced reliance on word entropy and sentence context to support speech comprehension, the results indicate an increased reliance on higher-level linguistic processing in late adulthood across the behavioral and neural levels. Consistent with previous behavioral and neural results^13,24,35^, these findings indicate that older adults might compensate for lower-level peripheral declines by using higher-level linguistic processing. This is supported by our evidence showing that increased word-level tracking can compensate for detrimental effects of a stronger distractor representation across the adult lifespan. Thus, an increased reliance on higher-level processing may serve as a strategy to maintain successful speech comprehension in challenging listening conditions across the adult lifespan^62,63^.

## Conclusions

In sum, we found a behaviorally relevant decline in attentional filtering across the adult lifespan, which might underlie speech comprehension difficulties in late adulthood. This lower-level impairment may be compensated for by an increased reliance on higher-level mechanisms at the behavioral and neural levels. Thus, older adults may increasingly rely on contextual cues provided in natural language to maintain speech comprehension abilities in late adulthood. These results provide implications for interventions targeting speech comprehension and social integration in late adulthood, arguing for enhancing higher-level cognitive processing to support compensational recruitment in speech comprehension.

## Materials and methods

### Participants

Participants aged 19–71 years were recruited from the participant database maintained at the Max Planck Institute for Human Cognitive and Brain Sciences, Leipzig, Germany. Parts of the current sample were used in ref. ^46^. Participants received monetary compensation of 12 € per hour for their participation. The study was approved by the ethics committee of the medical faculty at Leipzig University (ethics vote: 032/24-ek). All participants provided written informed consent before starting the experiment. Inclusion criteria included age-appropriate psychosocial functioning as well as normal hearing. Psychosocial functioning was confirmed by a score of 27 or higher on the Mini-Mental State Examination^64^. For normal hearing, the criterion was a pure tone threshold below or equal to 25 dB HL across frequencies from 250 to 8000 Hz in at least one ear. 4 additional participants were excluded after screening due to insufficient hearing performance. The mean pure tone threshold for the remaining participants’ better ear in our sample was *M* = 8.12 dB HL (range = −4 to 25 dB HL). The full audiometric results for the included participants can be retrieved from Fig. S1. All participants were German native speakers, right-handed, and reported being neurologically and psychiatrically healthy. The final sample comprised 63 participants (35 women) aged 19–71 years (*M* = 43.75, SD = 17.15).

### Procedure

The experiment was conducted in a sound-proof, electromagnetically shielded booth. Stimuli were presented using Koss KSC75 ear-clip headphones, and speech recordings were administered using a condenser microphone (Rode NT55) at 44100 Hz. The experiment was administered by the Psychophysics Toolbox Version 3 running on MATLAB on a Windows machine^65,66^. Participants completed 240 trials (12 blocks of 20 sentences). Participants completed 240 trials (12 blocks of 20) in a selective auditory attention paradigm (Fig. 1; see ref. ^46^ for full protocol details). Each trial presented two concurrent sentences: a male target sentence to be attended and a female distractor sentence to be ignored. After listening to the sentences and a brief period of 1 s to avoid EEG artifacts, participants were instructed to repeat as much of the target sentence as possible. If participants only understood parts of the sentences, they repeated only these parts. The behavioral paradigm is schematically shown in Fig. 1. The experimental procedure is described in full detail in ref. ^46^.

To control behavioral performance for peripheral differences in hearing abilities, experiment difficulty was adjusted individually. SRTs were measured using an adaptive staircase procedure (5 dB initial TDR, 6 dB initial step size, 0.85 step-size scaling per turning point)^67,68^ including 20 additional trials prior to the main experiment. If participants correctly repeated the target sentence, the intensity of the target sentence was decreased, whereas intensity was increased when participants missed at least one word. Distractor intensity was held constant at 70 dB SPL. Individual SRTs were calculated as the average of the TDR ratios preceding the final 5 turning points. The mean SRT across participants was *M* = −7.70(SD = 1.36).

Sentences were presented at six individually adjusted target-to-distractor ratios [TDR; −2, −1, 0, 1, 2, 10 dB relative to individual 50% SRTs]. The last condition served as a control for attention and working memory. Control trials were excluded from the neural analyses, but their mean accuracy was included as a control variable in the statistical analysis. The mean accuracy in the control trials was satisfyingly high in all participants (*M* = 97%, range = [91%, 99.5%]). Participants additionally listened to one block of word lists composed of unrelated nouns presented at 0 dB TDR relative to their SRTs.

### Stimuli

Stimuli were recorded at 44,100 Hz on a high-quality condenser microphone (Rode NT55) in a sound-proof booth. The distractor sentences started 0.5 s prior and lasted for a minimum of 0.3 s longer than the target sentences to ensure a complete masking of the target stream. Accordingly, the target sentences were composed of 5–7 words and lasted for 2–4 s. The distractor sentences comprised 7–10 words and lasted for 3–5 s. The sentence material was retrieved from a German sentence corpus^69^ after filtering for the suitable sentence length and excluding sentences that contained names or very unusual words. Additionally, similar suitable sentences were created by GPT-3.5. The prompts used to create the sentences can be retrieved from Table S2. All results remained stable after adding a control predictor for the sentence origin (corpus versus GPT, see Table S3). All sentences were loudness normalized to −23 dB LUFS using pyloudnorm^70^.

### EEG data acquisition

EEG data were recorded at 1000 Hz in a soundproof, electromagnetically shielded booth using a 68-channel REFA8 amplifier (TMSi, Oldenzaal, the Netherlands). Continuous EEG was acquired from 63 Ag/AgCl electrodes mounted in an ANT Neuro Waveguard cap (10-20 system, sternum ground) alongside two external mastoid references (A1, A2). Horizontal and vertical eye movements were tracked using bipolar electrooculogram (EOG) electrodes placed at the outer sides of both eyes and at the top and bottom of the right eye. Signals were continuously monitored for quality during the session.

### EEG preprocessing

EEG data were preprocessed using MNE-Python^71^ in accordance with established guidelines for TRF analyses^72^. Signals were bandpass-filtered between 0.5 and 15 Hz and re-referenced to the average of the mastoids. Channels displaying excessive variance relative to neighboring channels were manually identified and removed. To attenuate ocular artifacts, independent component analysis (ICA; 15 components) was performed, with artifactual components identified and rejected automatically based on their correlation with the EOG channels. Rejected bad channels were subsequently interpolated using spherical splines^73^. Finally, signals were downsampled to 128 Hz to optimize computation time during TRF estimation and epoched from target sentence onset to offset.

### Features

#### Acoustic features

Speech features were extracted separately for the target and distractor streams. Auditory spectrograms were generated using the naplib-python package^74^, implementing a cochlear filter bank of 128 logarithmically spaced constant-*Q* filters paired with a hair-cell model to simulate human peripheral auditory processing. Broadband acoustic envelopes were computed by averaging energy across all 128 spectrogram channels. Envelope derivatives were calculated by taking the half-wave rectified derivative of the broadband envelope^31^.

Phoneme and word onsets were obtained through forced alignment using WebMAUS Basic^75,76^. To isolate sentence-onset responses from ongoing linguistic processing, the first word onset of each sentence was modeled as a standalone predictor, with general word-level predictors containing only subsequent word onsets.

#### Word-level features

Word frequencies were obtained from the SUBTLEX-DE corpus^77^ and defined as the logarithmic occurrence rate per million words.

Information-theoretic metrics (surprisal and entropy) were estimated using german-gpt2^78^, an autoregressive causal language model based on the GPT-2 architecture and trained on German corpora. Word surprisal quantifies the unexpectedness of a word *w*_*i*_ given its preceding sentence context^79,80^:

$$surprisa{l}_{i}=-{\log }_{2}(p({w}_{i}| context))$$

with $\left(p({w}_{i}| {\mathrm{context}})\right.$ being the word probability given the preceding sentence context derived from german-gpt2^78^.

Word entropy is defined as the uncertainty of the upcoming word given the previous sentence context from the previous word *w*_*i*−1_ to the first word in the sentence *w*_*i*−*n*_.

$${{\mathrm{entropy}}}_{i}=-\mathop{\sum}\limits_{k}P({w}_{k}| {w}_{i-1}...{w}_{i-n}) * {{\mathrm{log}}}_{2}(P({w}_{k}| {w}_{i-1}...{w}_{i-n}))$$

Because german-gpt2 uses Byte-Pair Encoding, words split into multiple subword tokens were aggregated by summing token-level surprisal and entropy values to yield word-level metrics. As with the acoustic-linguistic features, word-level predictors excluded the first word of each sentence to control for sentence-onset responses.

#### Phoneme-level features

Phoneme surprisal and entropy were modeled to capture probabilistic processing dynamics at the sub-lexical level. Phoneme surprisal quantifies the unexpectedness of a phoneme *i* given the preceding phonemes within the same word, weighted by word frequency. Phoneme surprisal was defined as the inverse conditional probability of each phoneme, given the preceding phonemes in the same word:

$${{surprisal}}_{i}=-{{log}}_{2}\left(\frac{{freq}({{cohort}}_{i})}{{freq}({{cohort}}_{i-1})}\right)$$

Cohort_*i*_ represents the set of candidate words matching the phonemic input up to phoneme_*i*_ and freq(cohort_*i*_) is the cumulative frequency of all words remaining in that cohort.

Phoneme entropy measures the uncertainty regarding the upcoming phoneme, defined over the probability distribution of candidate words in the active cohort:

$${{entropy}}_{i}=-\mathop{\sum}\limits_{{word}}^{{{cohort}}_{i}}{p}_{{word}} * {{log}}_{2}({p}_{{word}})$$

where *p*_word_ denotes the relative frequency-based probability of word *w* within the cohort. To dissociate sub-lexical processing from word-level onset responses, word-initial phonemes were omitted from all sub-lexical feature matrices. Finally, all features were min-max normalized prior to TRF estimation to preserve natural zero-baselines for sparse regression modeling.

### Speaker characteristics

As mentioned above, target and distractor sentences were presented by a male and a female voice, respectively. There was only one male and one female speaker across the full experiment. To avoid introducing an age-related confound due to the greater severity of age-related hearing loss in higher frequencies, we refrained from randomizing the assignment of the male and female speakers to the target and distractor roles. This was done to prevent confounding effects of the speakers’ pitch height, potentially dampening the effects. To control for the differences introduced by the speakers, the analysis was controlled for fundamental frequency. Fundamental frequency was calculated using the pYIN algorithm^81,82^ as implemented in the Python package librosa^83^.

### TRF models

Neural encoding of acoustic and linguistic features was quantified via forward multivariate TRFs implemented in mTRFpy^84^ using linear regression with ridge regularization. The TRF model characterizes the linear mapping from stimulus features to continuous EEG responses over defined latency windows^72^. Model weights were estimated across time lags from -100 to 800 ms relative to event onset. Predicted EEG signals were reconstructed via convolution of the TRF kernels with stimulus feature matrices. Prediction accuracy was defined as the Pearson correlation between actual and reconstructed EEG signals. This correlation was used to quantify the degree of neural tracking for each feature set.

TRF models were trained within participants. We used a 90-10 cross-validation with 50 randomly sampled folds following recent recommendations for evaluating brain decoding models^85^. For each fold, 20 trials were randomly selected to comprise the test set. Data from these trials was held out for model training. We obtained trial-wise model fits for each individual trial from the TRF models trained across trials. Each trial appeared in the test set five times. The mean of all five cross-validated model fits was used for all subsequent analyses. Model fit was defined as the Pearson correlation between predicted and observed EEG across the full trial, averaged across sensors. The results did not depend on the exact choice of the cross-validation procedure (see Figs. S3 and S4 for a reproduction of the results using a 10-fold cross-validation with independent folds).

Higher-level linguistic features in continuous speech are inherently collinear with lower-level acoustic properties. To isolate the unique neural tracking of linguistic information, we controlled for acoustic variance by residualizing lower-level acoustic responses from the EEG signals prior to higher-level analyses^34,86^. For the residualization, a separate TRF analysis was conducted predicting the EEG signals from all acoustic variables with a 10-fold cross-validation. These acoustic model predictions were then subtracted from the original EEG recordings, yielding residualized EEG responses free of lower-level acoustic responses. All analyses involving higher-level probabilistic features were conducted on these residualized EEG responses.

The regularization parameter was fitted separately for lower-level and higher-level models^87^. Fitting individual regularization parameters per participant led to overfitting in the acoustic analyses, and we therefore opted to fit the regularization parameters across participants. The optimal regularization parameter was determined based on the median across participants, as the mean regularization was strongly driven by a small number of outliers displaying very high regularization parameters. There was no correlation between age and the individual regularization parameters, suggesting that the model fitting procedure did not bias the age effects.

### Statistical inference

To evaluate the statistical significance of TRF model fits, we used a non-parametric permutation approach. Predictors were grouped into feature sets (summarized in Table 2) to mitigate multicollinearity during regression modeling. Statistical inference was derived across 599 shuffling iterations per model variant^88^. In each iteration, the target feature set was permuted while holding all other input features and model hyperparameters constant. Permutation strategies were tailored to the temporal structure of each feature class. For acoustic models, trial labels were randomly shuffled across conditions. For higher-level linguistic models, we adopted a more conservative permutation scheme: feature values were randomly shuffled across events while preserving their onset times.

**Table 2:** Feature groups

Model variant	Shuffled features
Acoustic	Envelope, envelope derivative
Onsets	Word onsets, phoneme onsets
Phoneme level	Phoneme surprisal, phoneme entropy
Word level	Word surprisal, word frequency, word entropy

Statistical significance was inferred by subtracting the mean shuffled model fits from the unshuffled model fits. Statistical inference was performed using a one-sided cluster-based permutation one-sample *t*-test with threshold-free cluster enhancement with 9999 permutations and a cluster-forming threshold of *α* = 0.05. Adjacency was computed using Delaunay triangulation of sensor positions, and the analysis was controlled for multiple comparisons separately for each feature and stream. Only the features displaying significant encoding performance in at least one sensor were used in the subsequent analyses. No significant tracking for the distractor higher-level features could be observed, indicating that these features do not show a generalizable effect on encoding performance. Thus, the distractor higher-level features were not used in any of the subsequent analyses, consistent with previous results on similar datasets^31^. In a previous study on the same data^46^, higher-level tracking for the distractor stream only emerged when isolating the incorrect trials.

#### Time-resolved encoding accuracy

To further disentangle the time scale of the word-level effects, we derived a time-resolved measure of encoding accuracy. In this analysis, the TRF was split into windows of 6 samples in steps of 3 samples. The TRF weights were set to zero outside of these windows, so that only certain sections of the TRF weights were used for the prediction of the EEG signal^89,90^.

#### Age effects on encoding accuracy

To investigate if the neural tracking of the investigated features increases with increasing age, we conducted multiple linear regression analyses predicting the subject-wise model fits from age. The analysis was controlled for the SRT and the mean accuracy in the main experiment and in the control condition by adding these variables as additional predictors. Due to the high correlation between age and hearing ability, as measured by the PTA, we added the orthogonalized PTA into the regression analyses. This means that only the share of variance in the PTA that could not be explained by age was added into the model. The correlations between age and the control variables are displayed in Fig. S2.

### Behavioral analysis

The speech recordings were automatically transcribed offline using Whisper^91^. Subsequently, the transcriptions were manually controlled. We then assessed the overlap between the transcribed response and the target sentence, marking each word as accurately repeated or inaccurate.

To investigate how lower-level acoustic and higher-level linguistic features influence sentence comprehension, word-by-word repetition accuracy was predicted from word surprisal, word entropy, and word audibility. Word audibility was operationalized as the glimpse rate, which measures the proportion of a target word that remains spectrotemporally audible despite energetic masking from the competing distractor stream^92^. Because acoustic masking fluctuates dynamically in competing speech, brief, unmasked speech segments ("glimpses") remain audible and can be integrated to support sentence comprehension. To quantify available glimpses per target word, we calculated the glimpse rate across each word boundary segment following^92^. This metric compares the spectrogram amplitudes of target and distractor speech streams. Specifically, for each target word segment, the glimpse rate was defined as the proportion of spectrotemporal bins in which the local target-to-distractor ratio exceeded a predefined threshold of −5 dB^92^.

To evaluate the effects of word surprisal, word entropy, word frequency, audibility, and their interactions on sentence comprehension, word-by-word repetition accuracy was modeled using a generalized linear mixed model with a logistic link function implemented in the lme4 package in R^93^. The analysis was controlled for word order, word length, sentence length (in words), TDR condition, SRT, orthogonalized PTA, and control accuracy by adding these variables as additional predictors. Random intercepts were included for participants and target sentences. Finally, to examine whether neural tracking predicted behavioral performance, trial-wise TRF model fits for all model variants were included as predictors in the logistic regression. We included interactions between age and all variables of interest (word surprisal, entropy, audibility, and TRF model fits). All variables were z-scored before entering the regression model. Only one mixed-effects model was used to investigate the effects of the speech features and the neural tracking estimates on behavioral comprehension performance. With regard to age, the original age values are used only for illustration purposes.

To assess potential multicollinearity among predictors, variance inflation factors were computed using the R package car^94^. All variance inflation factors ranged between 1.00 and 3.80, indicating that multicollinearity did not distort model estimates^95^. The highest variance inflation factors were observed for word surprisal and word frequency. However, despite their substantial correlation (*r* = −0.67), we assume that there was sufficient non-redundant information in both predictor variables. The full model explained 20.7% of variance in comprehension considering fixed effects only, and 50.4% of variance if random effects were taken into account.

To quantify each individual’s gain from sentence context, mean performance in the main experiment was compared with the mean performance in an additional block of word lists. The words contained in the lists were selected from all nouns appearing in the sDeWac corpus^96^ at least 1000 times, with a length between 5 and 11 letters. We furthermore wanted to avoid involuntary similarities between nouns in the word lists, as this could have created a sense of coherence while listening to the lists of words. Therefore, we chose the nouns that on average had the lowest similarities to all other candidate nouns. As a measure of semantic similarity, we used cosine similarity among the corresponding word vectors in fastText, a widely used word embedding model^97^. To investigate age effects, we predicted the standardized difference scores from age, controlling for SRT, orthogonalized PTA, control accuracy, and mean accuracy in the main experiment using multiple linear regression.

#### Compensational effects of higher-level tracking

In an exploratory analysis, we tested if word-level tracking can compensate for increased susceptibility to distraction. Therefore, we calculated the mean accuracy for each participant. This accuracy was predicted from the mean encoding accuracy for word-level target features and for distractor onsets and acoustics. We included three-way interactions between age, word-level target tracking, and distractor onsets and acoustic tracking. The analysis was controlled for the SRT, orthogonalized PTA, and the accuracy in the control condition, serving as a proxy for performance under highly intelligible listening conditions, likely driven by working memory. *P*-values were corrected for multiple comparisons using FDR.

### Statistics and reproducibility

Statistical analyses were conducted using standard toolboxes in R and Python. Analyses were conducted at the participant level (significance of TRF fits, age effects on TRF fits) and on the word level (neuro-behavioral correlations). For analyses of word- and trial-level data, generalized linear mixed-effects models were used to account for the repeated observations within participants and sentences, with random intercepts for participants and sentences where applicable.

Each participant contributed a single independent biological replicate. The individual trials contributed by each participant were treated as repeated measurements rather than independent biological replicates. The final sample comprised 63 participants. The sample size was based on previous studies investigating age-related differences in speech perception and neural tracking using EEG. All participants completed the same experimental paradigm, comprising 240 experimental trials. Exact *p*-values, effect sizes, confidence intervals where applicable, and detailed descriptions of all statistical procedures are reported in the manuscript.

### Reporting summary

Further information on research design is available in the Nature Portfolio Reporting Summary linked to this article.

## Supplementary information

Transparent Peer Review file

Supplementary Material

Description of Additional Supplementary Files

Supplementary data 1

Reporting Summary

## Data Availability

Preprocessed EEG data can be retrieved from https://doi.org/10.17605/OSF.IO/N7HZ. Behavioral data are available along with the code at: https://doi.org/10.5281/zenodo.22068474. Source data are provided with this paper.

## Code availability

All custom code underlying the conclusions can be retrieved from ref. ^98^ at https://doi.org/10.5281/zenodo.22068474.
