# Supplementary material for: Linguistic information may compensate for age-related decline in attentional filtering
Source: Commun Biol. 2026 Sep 22;9:1236. doi: 10.1038/s42003-026-10999-y (PMC13598105; doi:10.1038/s42003-026-10999-y)
Supplement: Supplementary file 1 [file 42003_2026_10999_MOESM1_ESM.pdf]

nature portfolio

Peer Review File

Linguistic information may compensate for age-related decline in attentional filtering

Corresponding Author: Ms Alice Vivien Barchet

This file contains all reviewer reports in order by version, followed by all author rebuttals in order by version.

Version 0:

Reviewer comments:

Reviewer #1

(Remarks to the Author)
Brief summary of the manuscript
In this study, the authors test whether age-related changes in speech comprehension in multitalker settings reflect altered attentional filtering and/or greater reliance on linguistic context. Sixty-three adults (19–71 years) performed a two-talker sentence task with individually adjusted target-to-distractor ratios based on each participant's speech reception threshold. Behavioral performance was scored at the word level and modeled as a function of audibility and language-model–derived predictability (surprisal/entropy). In parallel, EEG temporal response functon(TRF) modeling was used to estimate neural tracking of target and distractor acoustic-onset features and target linguistic features, and trial-wise TRF-derived metrics were related to trial-wise comprehension.
Overall assessment
The question is timely and relevant, and the combination of individualized difficulty adjustment, word-level behavioral modeling, and TRF-based speech tracking is appropriate for addressing it. The work should be of interest to readers studying auditory cognition, aging, and neural encoding of speech in complex environments. The main issues that currently limit the strength of the conclusions are methodological transparency and robustness of the central neural predictors: the derivation of trial-wise TRF "model-fit" measures is not specified in a way that allows independent evaluation, the linguistic TRF results rely on a non–crossvalidated residualization step that needs a robustness check, and the TRF inference procedure requires clearer description (and likely stronger null estimation). In addition, sensitivity analyses are needed to address plausible confounds introduced by fixed mapping of attentional role to speaker identity and by the mixed stimulus/predictor pipeline (corpus plus GPTgenerated sentences; GPT-2–derived surprisal/entropy). Finally, the "compensation" framing should be calibrated to the correlational nature of the evidence unless supported by a targeted interaction analysis consistent with compensation.
Major Concerns
L127–133; L436–439 - Trial-wise TRF model-fit metric is not defined in a checkable way.
Issue: Cross-validation is described, but it is unclear how a single-trial "model-fit" value is computed when trials appear in multiple test folds, and how sensors/time windows enter the score.
Recommendation: Define the exact per-trial score (metric, time window, sensor aggregation), specify how repeated estimates for the same trial are combined, and report a simple stability check (for example compute trial-wise scores from two independent sets of folds and show they correlate).
L440–447 - Linguistic TRFs rely on non-cross-validated acoustic residualization.
Issue: Subtracting acoustic predictions fit without cross-validation makes the resulting "linguistic-only" signal hard to interpret and may remove shared variance or fitted noise.
Recommendation: Add one robustness analysis: either fit acoustics and linguistic predictors jointly in one TRF model (with regularization/variance partitioning), or redo the residualization using cross-validated acoustic predictions (predict held-out EEG, then subtract).

L455–456; L462–466 - TRF inference needs clearer specification and likely stronger null estimation.
Issue: The null distribution is based on 50 shuffles, and the cluster procedure is not described in enough detail (adjacency/cluster statistic). It is also unclear what multiple-comparison family is being corrected.
Recommendation: Increase shuffle iterations (or show results are unchanged with a higher number), state the sensor adjacency and cluster statistic used, and specify what set of tests is corrected together (streams × predictors × conditions).
L345–347 - GPT-3.5-generated sentences are included but not characterized; stimulus-source confound not tested.
Issue: The manuscript does not report how many GPT-generated sentences were used or whether they differ from corpus sentences in length or surprisal/entropy distributions, which could influence linguistic effects.
Recommendation;: Report the number/proportion of GPT-generated sentences and the selection/validation procedure; rerun the key effects excluding GPT items and/or include a "GPT vs corpus" indicator covariate (and report whether key effects change).
L385–394 - Surprisal/entropy computation (German GPT-2) is under-specified; sensitivity not shown.
Issue: Key implementation details that affect surprisal/entropy are not provided (checkpoint, tokenization, context definition, entropy computation).
Recommendation: Specify the exact model/checkpoint and computation details, then run a minimal sensitivity check (for example an alternative checkpoint or small implementation variant) to show the main linguistic effects keep the same direction.
L309–312; L411–416 - Speaker identity is confounded with attentional role (male target, female distractor).
Issue: Target versus distractor effects are inseparable from voice identity, limiting how strongly results can be interpreted as attentional filtering/suppression.
Recommendation: Tone down role-specific causal language and add a control showing effects are not explained by voice separability (for example include a trial-wise separability metric beyond F0 and show the age effect on distractor-onset tracking remains).
Minor Concerns
L1; L8–19; L172–186; L210–216; L276–281; L283–290 - Compensation framing exceeds what the current analyses establish.
Issue: The manuscript states that linguistic tracking compensates/offsets age-related distractor effects, but the evidence shown is correlational.
Recommendation: Soften wording to reflect correlation, or add a focused analysis that is consistent with compensation (for example an interaction showing that linguistic tracking predicts comprehension more strongly at harder TDRs and/or when distractor-onset tracking is high). Indicate whether this analysis is confirmatory or exploratory.
Literature Suggestions
- L49–51 (compensation vs inefficiency/noise interpretation of stronger tracking): add Henry et al. (2017).
- L239–242 (link between higher-level linguistic/semantic tracking and comprehension): add Broderick et al. (2018) and Weissbart et al. (2020).
- L276–281 (mechanistic framing for "compensation" under degraded input): add Rönnberg et al. (2013) and Pichora-Fuller et al. (2016).
- L385–389 (theoretical grounding for surprisal as a predictor): add Levy (2008) and Hale (2001).
- L440–446 (multi-feature TRF methodology / feature separation): add Crosse et al. (2016) and Di Liberto et al. (2015).
References mentioned
Broderick, M. P., Anderson, A. J., Di Liberto, G. M., Crosse, M. J., & Lalor, E. C. (2018). Electrophysiological correlates of semantic dissimilarity reflect the comprehension of natural, narrative speech. Current Biology, 28(5), 803–809.e3. https://doi.org/10.1016/j.cub.2018.01.080
Crosse, M. J., Di Liberto, G. M., Bednar, A., & Lalor, E. C. (2016). The multivariate temporal response functon(mTRF) toolbox: A MATLAB toolbox for relating neural signals to continuous stimuli. Frontiers in Human Neuroscience, 10, 604. https://doi.org/10.3389/fnhum.2016.00604
Di Liberto, G. M., O'Sullivan, J. A., & Lalor, E. C. (2015). Low-frequency cortical entrainment to speech reflects phoneme-level processing. Current Biology, 25(19), 2457–2465. https://doi.org/10.1016/j.cub.2015.08.030
Hale, J. (2001). A probabilistic Earley parser as a psycholinguistic model. In Proceedings of NAACL (pp. 1–8). Association for Computational Linguistics.
Henry, M. J., Herrmann, B., Kunke, D., & Obleser, J. (2017). Aging affects the balance of neural entrainment and top-down neural modulation in the listening brain. Nature Communications, 8, 15801. https://doi.org/10.1038/ncomms15801
Levy, R. (2008). Expectation-based syntactic comprehension. Cognition, 106(3), 1126–1177. https://doi.org/10.1016/j.cognition.2007.05.006

Pichora-Fuller, M. K., Kramer, S. E., Eckert, M. A., Edwards, B., Hornsby, B. W. Y., Humes, L. E., Lemke, U., Lunner, T., Matthen, M., Mackersie, C. L., Naylor, G., Phillips, N. A., Richter, M., Rudner, M., Sommers, M. S., Tremblay, K. L., & Wingfield, A. (2016). Hearing impairment and cognitive energy: The Framework for Understanding Effortful Listening (FUEL). Ear and Hearing, 37(Suppl 1), 5S–27S. https://doi.org/10.1097/AUD.0000000000000312
Rönnberg, J., Lunner, T., Zekveld, A., Sörqvist, P., Danielsson, H., Lyxell, B., Dahlström, Ö., Signoret, C., Stenfelt, S., Pichora-Fuller, M. K., & Rudner, M. (2013). The Ease of Language Understanding (ELU) model: Theoretical, empirical, and clinical advances. Frontiers in Systems Neuroscience, 7, 31. https://doi.org/10.3389/fnsys.2013.00031
Weissbart, H., Kandylaki, K. D., & Reichenbach, T. (2020). Cortical tracking of surprisal during continuous speech comprehension. Journal of Cognitive Neuroscience, 32(1), 155–166. https://doi.org/10.1162/jocn_a_01467

Reviewer #2

(Remarks to the Author)
I co-reviewed this manuscript with one of the reviewers who provided the listed reports. This is part of the Communications Biology initiative to facilitate training in peer review and to provide appropriate recognition for Early Career Researchers who co-review manuscripts.

Reviewer #3

(Remarks to the Author)
This paper highlights key predictors that may contribute to better speech-in-noise listening based on age-related changes. This paper fills gaps in our understanding between attention and speech processing as a function of age. One of the most valuable contributions of this work is including both behavior and speech tracking at the individual sentence level (most related work only has much coarser behavioral information).

The focus of this paper is to understand the age-related compensatory mechanisms in relation to listening to competing speakers. This paper uses EEG, along with behavioral data, to identify which predictors are modulated by compensatory mechanisms and the processing of which speech feature may be affected by age. The paper describes three main results.

Behaviorally, subjects show comprehension benefits from word uncertainty (as measured by lexical entropy) for all ages, but even more so for older adults. Neurally, they first show that comprehension is positively correlated with enhanced representations of the distractor at the acoustic level, and also correlated with enhanced representations of the distractor at the acoustic level, and of the target at the level of phoneme and of the target at the level of phoneme & word onset and phoneme & word statistics/context.

Surprisingly, no overall main effect of age was seen, either behaviorally or neurally.

Also, the authors' creative use of graphs to visually explain interaction effects (such as the rightmost graph of Figure 2C) is greatly appreciated. Interaction terms can be very tricky to interpret/explain, and the use of such graphs is very helpful.

Major Comments:

Several of the most critical definitions / use of terminology are very confusing and make it difficult to interpret the results. Among the most critical:
The acoustic predictors include both envelope and envelope onsets, but the term "onset" is widely used throughout the paper and does not refer to acoustic onset predictors.
The term "onset" as most commonly used in the paper applies to word- and phoneme-level onsets. Since words and phoneme use/statistics is language dependent, these are linguistic predictors. The authors do not group them with other linguistic predictors but with the acoustic predictors mentioned above, all together described as "lower-level". This is not problematic by itself, but then that group containing both is often described as containing only non-linguistic predictors, which is both confusing and incorrect.
The group called "higher-level" is entirely made of higher level linguistic predictors, which is fine, but the predictors of this group also referred to as "the linguistic predictors", which is confusing and incorrect since the linguistic onsets describe above are linguistic predictors but are not in this group.
Cleaning up these, and related, usages of terminology is most critical.

Task difficulty was individually adjusted based on the individual speech reception threshold (SRT) to control for effects of peripheral constraints. This is an important component of the experiment, but almost no information is given regrading how this was applied to the subjects. In particular, given the surprising finding that no overall main effect of age was seen for comprehension, is it possible that the expected main age effect was negated by customizing SRT? If so, this is important to know, but impossible for the reader to see given the lack of SRT results (especially SRT x age). Even if not, addressing the lack of overall main effect of age would be important regardless.

In a related context, regarding Controlling for Correlated Variables (Page 11, Line 178/179; Page 24, Line 476; Page 25,

Line 494): How was the analysis controlled for these variables (including SRT)? It looks like the only thing described is PTA (Line 478).

The audibility measure is never defined. Is it the same thing as the amount of available glimpses? Regardless such a critical measure should be carefully defined.

Lower-Level Comments:

Page 5, Lines 96-99; Page 6, Figure 1C; Page 14, Line 224, and others
"...word entropy had a stronger positive effect on comprehension accuracy."
Would the authors please expand on this phenomenon, explanation and interpretation? It seems counterintuitive that, regardless of age, greater uncertainty leads to better predicted accuracy. Addressing this confusion (even in the results section) would benefit the reader. Additionally, it seems that figure 1C could also be alternatively interpreted as that word certainty has a stronger negative effect on older adults (which seems like a more direct interpretation), and more guidance as to why the reader should not take that interpretation would be helpful.

TRF Calculation: It seems that two separate mTRF sets were calculated, one for the lower-level predictors and one for the higher-level predictors (using the residuals of the first set). Why were they not all calculated as an mTRF set that combines all acoustic and linguistic predictors simultaneously? Using the two stage method runs the risk of removing responses due to higher-level that have some correlation with lower level predictors.

Multicolinearity: Page 25, Line 494, Line 497-498
"The analysis was controlled for word frequency, sentence length (in words), …. We included interactions between age and all variables of interest (word surprisal, entropy, and audibility, TRF model fits)." This goes back to the previous comment about how the analysis was controlled for these variables. In Table 1, word frequency is used as a fixed effect, but it is not mentioned in the variables of interest. If word frequency should be included in the variables of interest, it is surprising that there were no issues with multicollinearity, as some issues between word surprisal and word frequency would be expected.

Page 11, Line 179 to Page 13, Line 183.
"Results revealed a significant interaction between the linguistic target and the onsets distractor encoding accuracies. This interaction indicates that the positive effect of linguistic target tracking on comprehension performance is stronger when the distractor onset tracking is increased (see Figure 3E). This indicates that stronger linguistic tracking might compensate for increased individual distractibility." This exploratory analysis was very interesting. However, since the model only looked at target linguistic tracking and distractor onset tracking, it is unclear whether this is the compensatory mechanism or if individuals who have an overall higher tracking of all predictors have better performance. One might add an additional variable in the model, such as distractor acoustic tracking, to verify that the compensatory mechanism is indeed the combination of these two variables rather than overall higher tracking.

Page 23, Line 445-448
"The regularization parameter was fitted separately for acoustic and linguistic models. The optimal regularization parameter was determined based on the median across participants, as the mean regularization was strongly driven by a small number of outliers displaying very high regularization parameters." Would you please expand why you used the same regularization parameter across participants? There are benefits to this (which would be good to hear expressed), but there is also the potential for concern that the same regularization parameter across such a large age range might bias the results in some way: e.g., if older adults have noisier baselines than younger adults. Were the subjects with very high regularization parameters the older adults?

Minor Comments:

Page 9, Figure 2C
The positive slope of the acoustic distractor seems at odds with the non-positive slopes of the onsets distractor. This is explained in the discussion but an acknowledgment of this and/or a quick explanation in results would greatly benefit the reader trying to parse the large number of results.

Page 14, Line 224-228
"[The positive effect of word entropy on comprehension performance] got stronger with increasing age, …. In line with this conclusion, older adults seemed to benefit more from overall sentence context, when comparing comprehension performance in sentences and word lists."

- It is not clear how the positive effect of word entropy is "in line with the conclusion" that older adults benefit from sentence context. These seem like opposite statements. Would you please explain?

Page 21, Line 406/407
"All features were min-max normalized to avoid negative values before entering the TRF."

- Why are negative values avoided? It is very common in TRF analysis to z-score predictors (and responses), which allows negative values. Also, min-max normalization is susceptible to random scaling artifacts due to min/max outliers (which could be avoided by, e.g., z-scoring or 5/95 percentile normalization).

Scaling for Figures
- Some variables look like they have been z-scored (audibility in Figure 1B), but this is not explained in the methods. If so, would you please add this to the methods?

“Trial-wise TRFs”
- It is unclear what is meant by trial-wise TRFs. Based on the methods, it seems like a single TRF is created (page 22), but “trial-wise” TRFs sounds like one TRF is created for each trial.

Page 21, Line 409
“As mentioned above, target and distractor sentences were presented by a male and a female voice, respectively.”
- It is unclear whether there are is a single female and single male speaker across all trials or if there are different female/male speakers.

Page 8, Table 1
- Should the interaction terms in the table have asterisks or colons? Based on the notation of R, an asterisk represents not only the interaction of the variables, but also includes the main effect; a colon would only represent the interaction itself.

Page 9, Figure 2 (and other locations, e.g. Page 11, Line 170)
“No significant effects of age on encoding accuracy strength were observed.”
- Please change this to “main effect of age” to avoid confusion regarding the presence of interaction terms with age.

Pages 9-10, Lines 153-154
“As displayed in Figure 3 A, the higher-level linguistic features were only significantly tracked for the target stream.”
- Figure 3A does not show this (i.e., it does not show that the higher-level linguistic features were not significantly tracked for the distractor stream).

Page 20, Line 389
- “N” is not defined
- This equation has an equation label as (1) but the other equations do not

Word TRF
- Currently, the “word TRF” is a combination of word entropy, surprisal, and frequency. Because there were behavioral results that saw an age effect with word entropy, we are curious to see what the effect of age is on the word entropy TRF.

Page 12, Figure 3D
- You might change the y-axis title to include that it is the late window encoding accuracy, to avoid confusion from Figure 3B.

Page 16, Lines 269-270
“This late processing window is typically associated with the processing of expectations and word identity “
- Please consider citing Ref. 44 here also, regarding the role of the late processing window and expectations and word identity; it is a more closely related case than the currently cited papers since the TRF framework was used there (like here).

Typos
Page 6, Figure A caption first line
- “by” → “of”?

Page 18, Line 322
- “latter” → “last”?

Page 24, Line 485
- capitalize Whisper

Page 24, Line 486
- possible typo, “controlled”

Version 1:

Reviewer comments:

Reviewer #1

(Remarks to the Author)
We thank the authors for their detailed response and for the revisions to the manuscript. Our assessment focuses on the points raised in our previous report.

The main methodological concerns from our report have largely been addressed. The trial-wise TRF model-fit measure is now described more clearly, and the added 10-fold cross-validation analysis with independent test folds provides a useful stability check. The residualization concern has also been addressed by rerunning the linguistic TRF analysis with cross-validated acoustic residualization. The removal of the phoneme-level linguistic fits after they no longer survived this

procedure is appropriate.

The TRF inference procedure is now better specified, with increased shuffle iterations and added details on TFCE, permutations, sensor adjacency, and correction scope.
The authors have also responded adequately to the stimulus- and design-related concerns. They now report the proportion of GPT-generated sentences, provide the prompts, and include a stimulus-source control model. The source effect suggests that GPT-generated sentences were easier to understand, but the main results appear stable after including this covariate; this should be reported transparently. The fixed speaker-role mapping remains a design limitation, but the added spectral-distance control is a reasonable post hoc check, and the rationale for not counterbalancing speaker roles is now clearer.

The reporting of the German GPT-2 surprisal and entropy predictors has improved. The Methods now specify the model, tokenizer, context definition, and token-to-word aggregation procedure. The authors did not run an alternative language-model sensitivity analysis, explaining that they relied on a pre-trained model and did not have access to alternative checkpoints. This does not fully address the sensitivity point, but we do not consider it a major remaining obstacle if the predictor computation is reproducible from the shared code.

One issue remains at the level of framing. The Results section now includes a clearer caveat that the compensation analysis is correlational and exploratory. However, the title and abstract still use stronger language than the evidence warrants, including “compensates,” “direct evidence,” “directly show,” and “offset.” We recommend softening the title and abstract to match the correlational nature of the evidence. Phrasing such as “is associated with,” “may support,” or “is consistent with a compensatory role” would better reflect the inferential strength of the analyses.

We also recommend one small wording correction in the Methods. The manuscript describes accuracy in the control condition as “serving as a proxy for working memory performance.” This measure is better described as a proxy for general task performance, attention, or performance under highly intelligible listening conditions, rather than working memory specifically.

In summary, the authors have addressed our main technical concerns. We do not see a need for another major revision from our side. The remaining changes are targeted wording revisions: calibrating the compensation language in the title and abstract, and revising the description of control-condition accuracy. Once these changes are made, we would consider the manuscript responsive to our previous review.

Reviewer #2

(Remarks to the Author)
I co-reviewed this manuscript with one of the reviewers who provided the listed reports. This is part of the Communications Biology initiative to facilitate training in peer review and to provide appropriate recognition for Early Career Researchers who co-review manuscripts.

Reviewer #3

(Remarks to the Author)
Thank you for your responsiveness to our review.
Only a few minor issues remain.

Page 8, Table 1.
Please consider adding the control variables for each model within this table, which mighty be helpful for the reader.

Page 12, Figure 3, Panel E.
Please change “Mean Accuracy” on y-axis to “Mean Comprehension Accuracy” to avoid confusion with encoding accuracy, and to better align with the text description in the figure caption.

Page 23, Lines 450-451, “Speaker characteristics”.
There are only 6 characteristics listed here, not 7.
Additionally, since there is only one male and one female speaker in this experiment, I’m not sure what the purpose is for including these characteristics at each word onset. Was this a separate predictor from word onset, or was each word onset multiplied by the mean rating before creating the acoustic model? If the former, I worry about the stimulus matrix not being a full-rank matrix.

Page 24, Line 477.
“hlhigher” should be “higher”

Page 24, Line 485.
delete “of”

Page 26. Lines 531-539.
Thank you for the definition on glimpse rate. Would you please define “audibility” more clearly within this context?

Open Access This Peer Review File is licensed under a Creative Commons Attribution 4.0 International License, which permits use, sharing, adaptation, distribution and reproduction in any medium or format, as long as you give appropriate credit to the original author(s) and the source, provide a link to the Creative Commons license, and indicate if changes were made.
In cases where reviewers are anonymous, credit should be given to 'Anonymous Referee' and the source.
The images or other third party material in this Peer Review File are included in the article's Creative Commons license, unless indicated otherwise in a credit line to the material. If material is not included in the article's Creative Commons license and your intended use is not permitted by statutory regulation or exceeds the permitted use, you will need to obtain permission directly from the copyright holder.
To view a copy of this license, visit https://creativecommons.org/licenses/by/4.0/

Manuscript COMMSBIO-26-1021
Response to Reviewers

Dear Editors,

We would like to thank you for the letter dated 19th March 2026, and the opportunity to submit a revised copy of the manuscript “Linguistic information compensates for age-related decline in attentional filtering” for consideration at Communications Biology.

We would like to thank the reviewers for their time and effort, their positive evaluation, and their constructive feedback. We comprehensively revised the manuscript along the following main points:

- We conducted sensitivity analyses with respect to the residualization procedure, the cross-validation, and the number of iterations.
- We included additional control variables in our analysis, including variables concerned with the origin of the sentences and the spectral distance between target and distractor sentences and revealed that the results did not change as a result of adding these predictors.
- We corrected the terminology related to the inaccurate comparison between linguistic and non-linguistic predictors, as suggested by Reviewer 3.
- We adjusted the method of controlling for differences in the pure tone average in our main regression model. Therefore, we now observe a negative effect of age on comprehension performance, which was expected by Reviewer 3.
- We clarified several methodological decisions.

We believe that these changes have resulted in an improved revised manuscript, which you will find uploaded alongside this document. We hope that our manuscript may ultimately be deemed suitable for Communications Biology. Additionally, we provide detailed point-by-point responses to the reviewers’ comments below. Reviewer comments are provided in bold font.

Reviewer #1 (Remarks to the Author):

Overall assessment

The question is timely and relevant, and the combination of individualized difficulty adjustment, word-level behavioral modeling, and TRF-based speech tracking is appropriate for addressing it. The work should be of interest to readers studying auditory cognition, aging, and neural encoding of speech in complex environments. The main issues that currently limit the strength of the conclusions are methodological transparency and robustness of the central neural predictors: the derivation of trial-wise TRF "model-fit" measures is not specified in a way that allows independent evaluation, the linguistic TRF results rely on a non–crossvalidated residualization step that needs a robustness check, and the TRF inference procedure requires clearer description (and likely stronger null estimation). In addition, sensitivity analyses are needed to address plausible confounds introduced by fixed mapping of attentional role to speaker identity and by the mixed stimulus/predictor pipeline (corpus plus GPTgenerated sentences; GPT-2–derived surprisal/entropy). Finally, the "compensation" framing should be calibrated to the correlational nature of the evidence unless supported by a targeted interaction analysis consistent with compensation.

Reply: We thank the reviewer for their positive assessment of our manuscript and their constructive feedback. We hope that we have addressed all concerns as detailed below.

Major Concerns

Issue 1:
L127–133; L436–439 - Trial-wise TRF model-fit metric is not defined in a checkable way.
Issue: Cross-validation is described, but it is unclear how a single-trial "model-fit" value is computed when trials appear in multiple test folds, and how sensors/time windows enter the score.

Recommendation: Define the exact per-trial score (metric, time window, sensor aggregation), specify how repeated estimates for the same trial are combined, and report a simple stability check (for example compute trial-wise scores from two independent sets of folds and show they correlate).

Reply: We added details on the summary procedure of the TRF model fits to the methods section. Additionally, we followed the reviewer's suggestion and conducted a stability check using a ten-fold cross-validation with independent test folds. This means that no trial was used twice in the test sets of the analysis. The correlation between the model fits obtained by our original method and the adjusted procedure was r = .98 for the lower-level

model and r = .96 for the higher-level model. All results could be replicated with the adjusted cross-validation. We added information about this analysis in the manuscript and added the adjusted Figures 2 and 3 into the supplementary material.

L. 467-471:

> Each trial appeared in the test set for 5 times. The mean of all 5 cross-validated model fits was used for all subsequent analyses. Model fit was defined as Pearson correlation between predicted and observed EEG across the full trial, averaged across sensors. The results did not depend on the exact choice of the cross-validation procedure (see Figures S3 and S4 for a reproduction of the results using a 10-fold cross-validation with independent folds).

Figure S3: Neural tracking and neuro-behavioral correlations for the lower-level features resulting from a 10-fold cross-validation

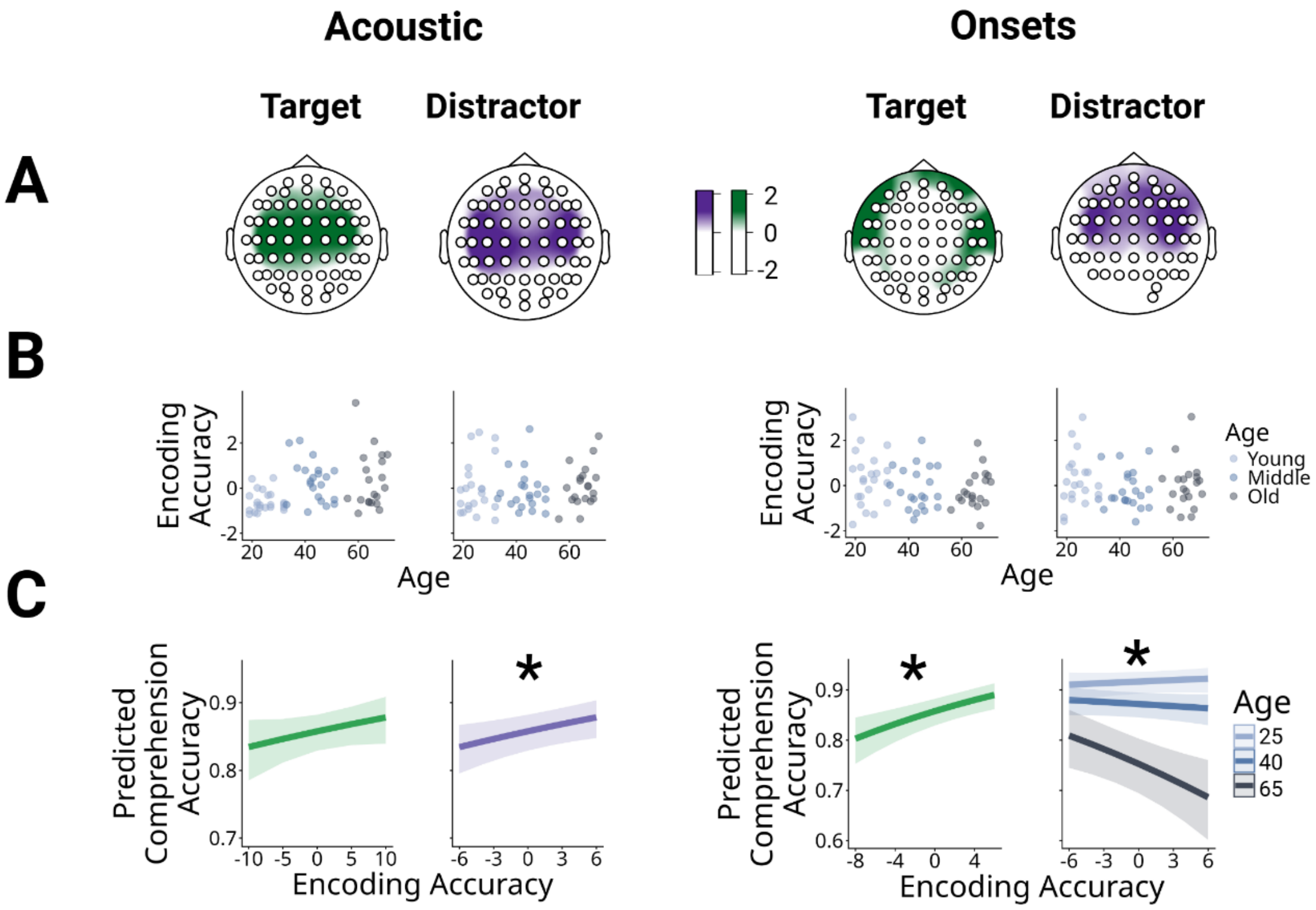

Figure S4: Neural tracking and neuro-behavioral correlations for the linguistic target features resulting from a 10-fold cross-validation

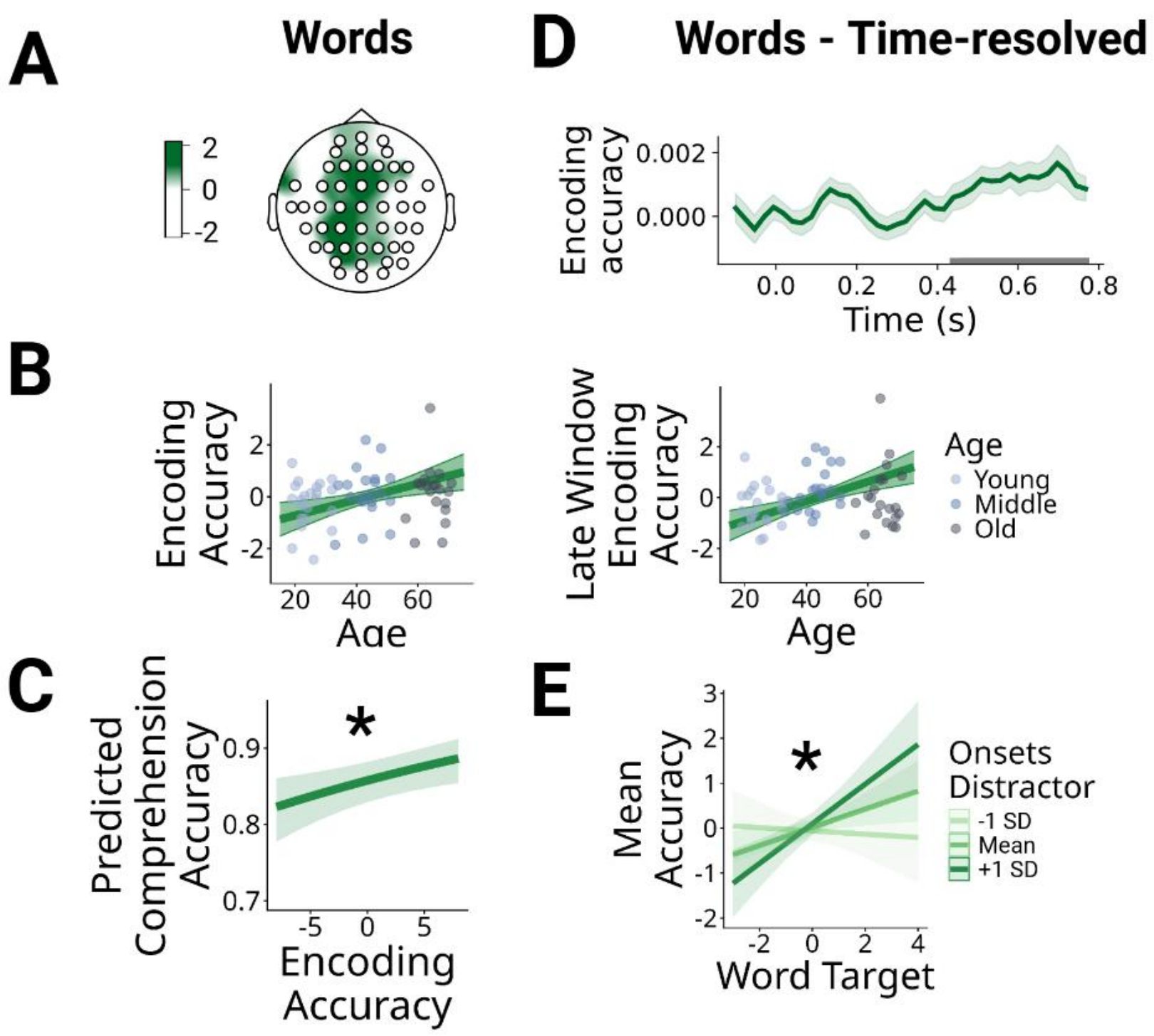

Issue 2:

L440–447 - Linguistic TRFs rely on non-cross-validated acoustic residualization. Issue: Subtracting acoustic predictions fit without cross-validation makes the resulting "linguistic-only" signal hard to interpret and may remove shared variance or fitted noise.

Recommendation: Add one robustness analysis: either fit acoustics and linguistic predictors jointly in one TRF model (with regularization/variance partitioning), or redo the residualization using cross-validated acoustic predictions (predict held-out EEG, then subtract).

Reply: We followed the reviewer's recommendation of running the residualization including a 10-fold cross-validation procedure. The main results remained unchanged and the correlation between the original and the adjusted model fits was r = .98. The only difference resulting from the adjusted residualization procedure was that the phoneme linguistic fits for the target sentences did not reach significance in the cluster-wise permutation tests. We therefore removed the phoneme linguistic fits from all further analyses. All other results remained unchanged. We append the adjusted version of Figure 3 below.

Figure 3: Neural tracking and neuro-behavioral correlations for the linguistic target features

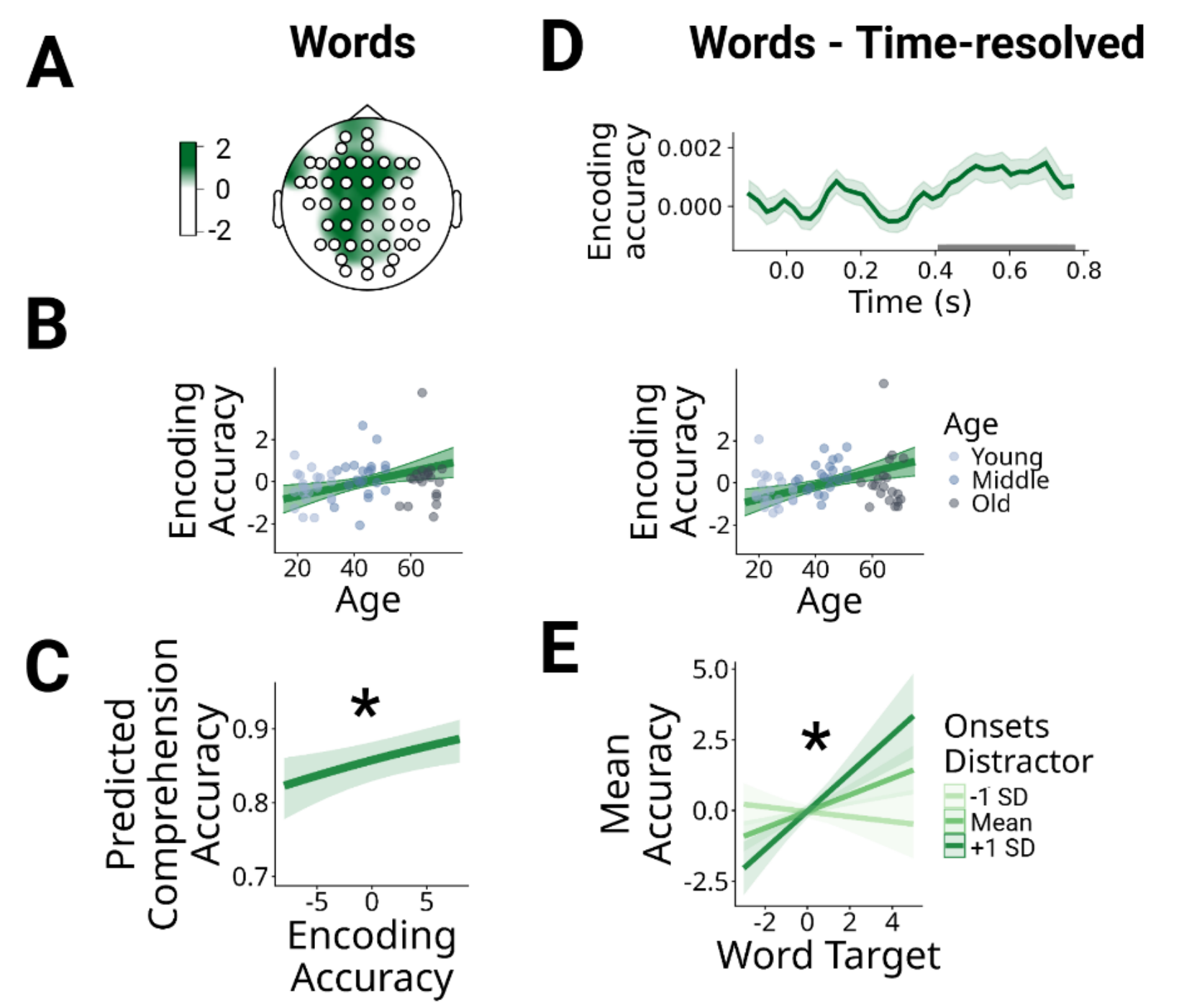

Issue 3:

L455–456; L462–466 - TRF inference needs clearer specification and likely stronger null estimation.

Issue: The null distribution is based on 50 shuffles, and the cluster procedure is not described in enough detail (adjacency/cluster statistic). It is also unclear what multiple-comparison family is being corrected.

Recommendation: Increase shuffle iterations (or show results are unchanged with a higher number), state the sensor adjacency and cluster statistic used, and specify what set of tests is corrected together (streams × predictors × conditions).

<u>Reply:</u> As suggested by the reviewer, we increased the shuffle iterations for both TRF models to 599 iterations, following recommendations for iteration-based statistical inference (Wilcox, 2001). The correlations between the original results with 50 iterations and the adjusted results was r = .99 for the lower-level model and r = .99 for the higher-level model. As for the adjusted residualization, the phoneme linguistic fits did not reach significance with the different null distribution. We believe that this version more accurately reflects the true null distribution and therefore report the adjusted version with more iterations in the main manuscript. We appended the adjusted versions of Figures 2 and 3 and Table 1 below.

Additionally, we added details about the TRF significance estimation in the methods section.

L. 492-494:

> "We conducted 599 shuffling iterations for each model variant following recommendations for iteration-based statistical inference [88]."

L. 500-503:

> "Statistical inference was performed using mass-univariate one-tailed one sample t-tests with threshold-free cluster enhancement with 9999 permutations and a cluster forming threshold of α = 0.05. Adjacency was computed using Delaunay triangulation of sensor positions and the analysis was controlled for multiple comparisons separately for each feature and stream."

Figure 2: Neural tracking and neuro-behavioral correlations for the lower-level features

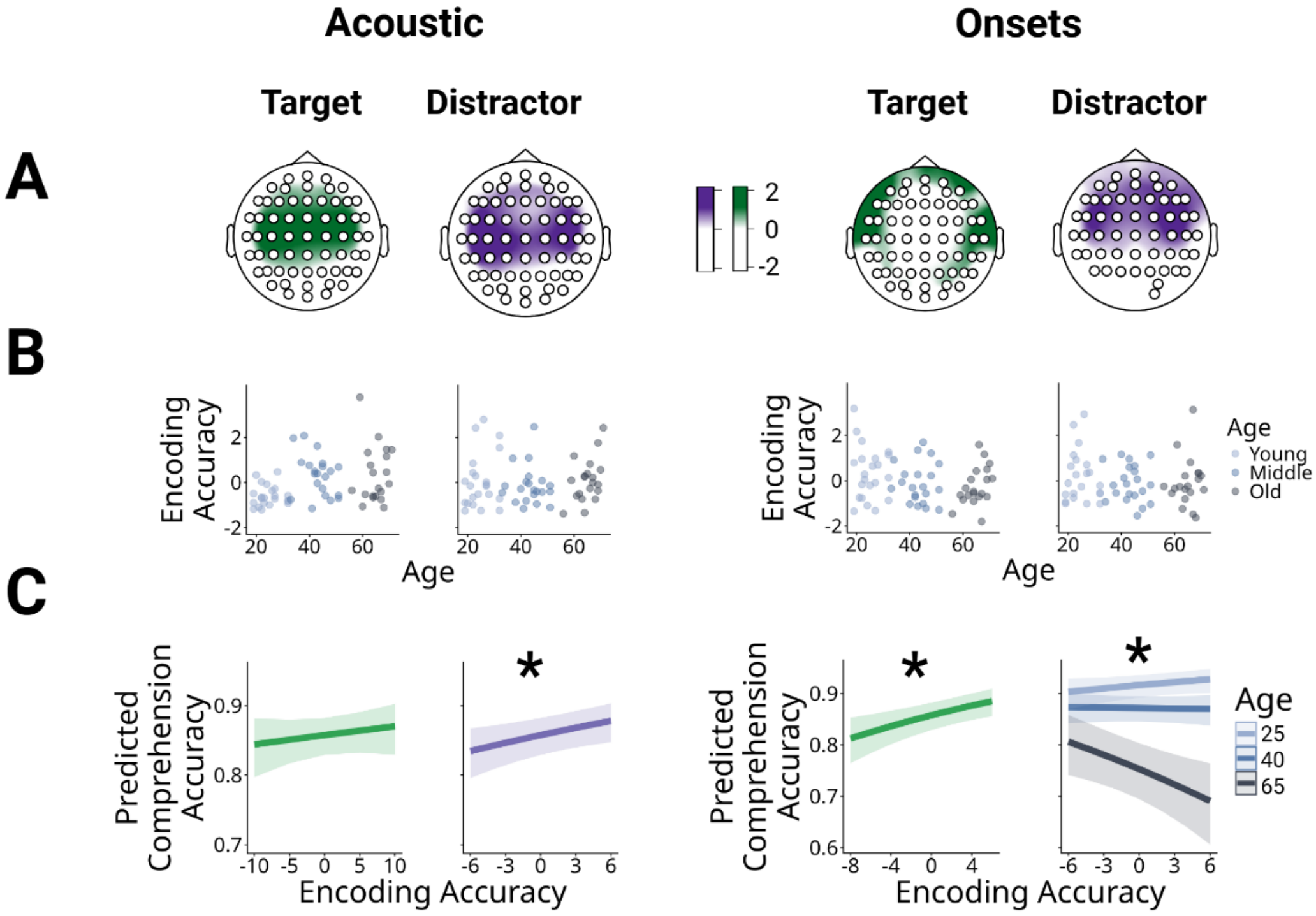

A: Topographies for the encoding accuracies. Significant electrodes in mass-univariate t-tests are marked by white dots. B: Age effects on encoding accuracy. No significant main effects of age on encoding accuracy strength were observed. C: Neuro-behavioral correlations and age interactions. Linear mixed model predictions of the effects of trial-wise neural encoding accuracy on comprehension accuracy. Distractor acoustic features and target onsets showed a positive relationship with comprehension accuracy. A significant interaction between the encoding model fits and age emerged for the

distractor onsets, suggesting that older adults' comprehension performance is more negatively influenced by the neural tracking of distractor features.

Figure 3: Neural tracking and neuro-behavioral correlations for the higher-level target features

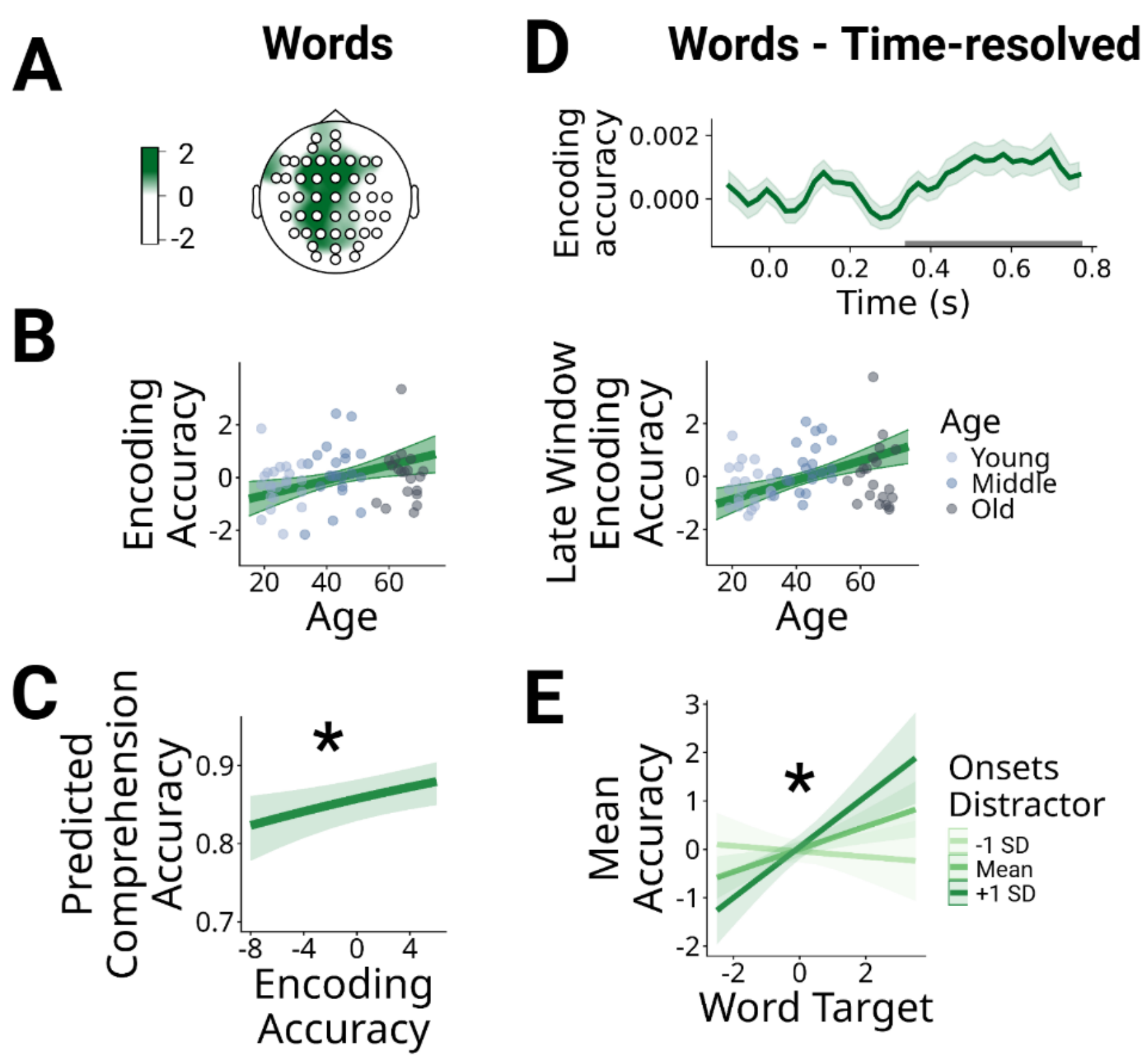

A: Topographies for the encoding accuracies. Significant electrodes in mass-univariate t-tests are marked by white dots. B: Age effects on encoding accuracy. Significant effects are marked by regression lines. There was a positive correlation between age and word-level encoding accuracy. C: Neuro-behavioral correlations. Linear mixed model predictions of the effects of trial-wise neural encoding accuracy on comprehension accuracy. Positive effects of word-level target tracking on comprehension accuracy. D: Time-resolved encoding accuracy for the target stream and age effect on encoding accuracy in a late window (400 - 800 ms). Grey bars indicate significant encoding performance. E: Word-level tracking may compensate for increased distraction. Significant interaction between the tracking of word-level target features and distractor onsets (Estimate = 0.03, SE = 0.01, t = 2.66, p = .023*). The positive effect of word-level target tracking on comprehension accuracy was larger at high levels of distractor onsets tracking, indicating a potential compensatory function of higher-level tracking.

Table 1: Model outputs predicting comprehension performance					
	Estimate	Std. Error	CI	z	p
**a) Behavioral effects**					
Age	-0.53	0.08	[-0.7, -0.37]	-6.43	<.001**
Audibility	0.41	0.01	[0.38, 0.44]	27.77	<.001**
Surprisal	-0.46	0.02	[-0.5, -0.42]	-22.29	<.001**
Audibility:Surprisal	0.17	0.02	[0.14, 0.2]	11.07	<.001**
Word frequency	0.15	0.02	[0.1, 0.19]	6.01	<.001**
Word entropy	0.21	0.02	[0.17, 0.24]	10.95	<.001**
Audibility:Age	0	0.01	[-0.03, 0.02]	-0.39	.756
Age:Surprisal	0	0.02	[-0.03, 0.03]	0.16	.905
Age:Entropy	0.04	0.02	[0.01, 0.07]	2.8	.009**
**b) Lower-level TRF fits**					
Acoustic target	0.01	0.01	[-0.01, 0.03]	0.89	.478
Acoustic distractor	0.03	0.01	[0.01, 0.05]	2.45	.022*
Onsets target	0.04	0.01	[0.02, 0.07]	3.35	.002**
Onsets distractor	-0.01	0.01	[-0.03, 0.01]	-0.86	.478
Age:Acoustic target	0	0.01	[-0.03, 0.02]	-0.08	.933
Age:Acoustic distractor	0.01	0.01	[-0.02, 0.03]	0.46	.730
Age:Onsets target	-0.01	0.01	[-0.04, 0.01]	-1.13	.370
Age:Onsets distractor	-0.03	0.01	[-0.06, -0.01]	-2.85	.008**
**c) Higher-level TRF fits**					
Word target	0.03	0.01	[0.01, 0.06]	2.7	.011*
Age:Word target	0.01	0.01	[-0.02, 0.03]	0.53	.698
**d) Control variables**					
Pure tone threshold	-0.07	0.02	[-0.1, -0.04]	-4.25	<.001**
Target-to-distractor Ratio	0.49	0.01	[0.47, 0.51]	55.89	<.001**
Wordlength	0.08	0.01	[0.07, 0.1]	11.64	<.001**
Word order	-0.13	0.01	[-0.15, -0.11]	-13.35	<.001**
Sentence length	-0.1	0.11	[-0.32, 0.12]	-0.9	.478
Control accuracy	0.24	0.07	[0.1, 0.38]	3.26	.002**
Speech Reception threshold	0.29	0.03	[0.23, 0.35]	9.11	<.001**

** p < .01, * p < .05 (FDR corrected)

Issue 4:

L345–347 - GPT-3.5-generated sentences are included but not characterized; stimulus-source confound not tested.

Issue: The manuscript does not report how many GPT-generated sentences were used or whether they differ from corpus sentences in length or surprisal/entropy distributions, which could influence linguistic effects.

Recommendation;: Report the number/proportion of GPT-generated sentences and the selection/validation procedure; rerun the key effects excluding GPT items and/or include a "GPT vs corpus" indicator covariate (and report whether key effects

change).

Reply: 58% of the sentences were generated by GPT. We added a supplementary table containing the prompts used to generate these sentences.

We additionally reran the mixed model analysis including a categorial predictor of the sentence origin (GPT versus corpus). While there seems to be a positive effect towards GPT-generated sentences being understood better, none of the results changed as a result of the inclusion of the predictor. We appended the adjusted model output table below for the reviewers' inspection.

Table S2: GPT Prompts for target sentence generation

**Prompts**
Give me 10 sentences with 5-7 words that could stem from a story.
Generate 10 everyday sentences with 5-7 words. Only use frequent words and do not use commas.
Variate the sentence structure. Do not use question marks or commas.
Generate more complex sentences with 5-7 words. Only use frequent words and do not use commas. Variate the sentence structure. Do not use question marks or commas.

Note. The same prompts were used for the distractor sentences, replacing 5-7 words with 7-10 words.

Model outputs including a predictor coding the sentence origin

	Estimate	Std. Error	CI	z	p
		**a) Behavioral Effects**			
Age	-0.53	0.08	[-0.7, -0.37]	-6.43	<.001**
Audibility	0.41	0.01	[0.38, 0.44]	27.81	<.001**
Surprisal	-0.46	0.02	[-0.5, -0.42]	-22.23	<.001**
Audibility:Surprisal	0.17	0.02	[0.14, 0.2]	11.06	<.001**
Word frequency	0.15	0.02	[0.1, 0.19]	6.07	<.001**
Word entropy	0.21	0.02	[0.17, 0.24]	10.94	<.001**
Audibility:Age	0	0.01	[-0.03, 0.02]	-0.39	.753
Age:Surprisal	0	0.02	[-0.03, 0.03]	0.16	.904
Age:Entropy	0.04	0.02	[0.01, 0.07]	2.8	.008**
		**b) Lower-level TRF fits**			
Acoustic target	0.01	0.01	[-0.01, 0.03]	0.89	.466
Acoustic distractor	0.03	0.01	[0.01, 0.05]	2.44	.022*
Onsets target	0.04	0.01	[0.02, 0.07]	3.35	.002**
Onsets distractor	-0.01	0.01	[-0.03, 0.01]	-0.87	.466
Age:Acoustic target	0	0.01	[-0.03, 0.02]	-0.09	.931
Age:Acoustic distractor	0.01	0.01	[-0.02, 0.03]	0.46	.724
Age:Onsets target	-0.01	0.01	[-0.04, 0.01]	-1.13	.342
Age:Onsets distractor	-0.03	0.01	[-0.06, -0.01]	-2.85	.008**
		**c) Higher-level TRF fits**			
Word target	0.03	0.01	[0.01, 0.06]	2.71	.011*
Age:Word target	0.01	0.01	[-0.02, 0.03]	0.53	.695
		**d) Control Variables**			
Pure tone threshold	-0.07	0.02	[-0.1, -0.04]	-4.25	<.001**
Target-to-distractor ratio	0.49	0.01	[0.47, 0.51]	55.9	<.001**
Wordlength	0.08	0.01	[0.07, 0.1]	11.65	<.001**
Word order	-0.13	0.01	[-0.15, -0.11]	-13.31	<.001**
Sentence length	-0.15	0.11	[-0.37, 0.06]	-1.4	.227
Control accuracy	0.24	0.07	[0.1, 0.38]	3.26	.002**
Speech reception threshold	0.29	0.03	[0.23, 0.35]	9.1	<.001**
Source [GPT]	0.65	0.17	[0.32, 0.97]	3.88	<.001**

** p < .01, * p < .05 (FDR corrected)

Issue 5:

L385–394 - Surprisal/entropy computation (German GPT-2) is under-specified; sensitivity not shown.

Issue: Key implementation details that affect surprisal/entropy are not provided (checkpoint, tokenization, context definition, entropy computation). Recommendation: Specify the exact model/checkpoint and computation details, then run a minimal sensitivity check (for example an alternative checkpoint or small implementation variant) to show the main linguistic effects keep the same direction.

Reply: We thank the reviewer for pointing out the need for a more detailed description of the surprisal and entropy computation. We have now expanded the Methods section accordingly, mentioning the model name and tokenizer and specifying the context used for surprisal and entropy computation. As we did not conduct the model training, but instead relied on a pre-trained model, we do not have access to alternative checkpoints or implementation variants to conduct a sensitivity analysis.

L. 407-408:

> "Word surprisal is defined as the inverse probability of each word given the preceding sentence context"

L. 410-413:

> "(...) derived from german-gpt2 [79]. This model is an autoregressive causal language model based on the GPT-2 architecture, trained on large-scale German text corpora. The model relies on a GPT-2 byte-pair encoding (BPE) tokenizer. This means that words may be split into multiple subword tokens."

L. 417-418:

> "Word-level surprisal and entropy were computed as the sum of token surprisals and entropies."

Issue 6:

L309–312; L411–416 - Speaker identity is confounded with attentional role (male target, female distractor).

Issue: Target versus distractor effects are inseparable from voice identity, limiting how strongly results can be interpreted as attentional filtering/suppression. Recommendation: Tone down role-specific causal language and add a control showing effects are not explained by voice separability (for example include a trial-wise separability metric beyond F0 and show the age effect on distractor-onset tracking remains).

Reply: We agree that the attentional role is confounded with the speaker identity and that this provides a potential limitation to the results. We, however, would like to emphasize that we controlled for additional speaker features beyond f0 in our TRF analyses. Additionally, as suggested by the reviewer, we calculated a trial-wise separability metric going beyond f0 based on the Euclidian distance between the target and distractor spectrograms. We added this distance metric into the linear mixed model. The predictor did not have a significant effect on comprehension performance. The results did not

change when adding the additional predictor. We appended the adjusted model output table for the reviewer's inspection below.

Additionally, we believe that randomizing the assignment of the attentional roles to the speakers would have introduced another confound, given the prevalence and stronger severity of age-related hearing loss at higher frequencies. We included this reasoning in the methods section.

L. 437-440:

> "To avoid introducing an age-related confound due to the stronger severity of age-related hearing loss in higher frequencies, we refrained from randomizing the assignment of the male and female speakers to the target and distractor roles. "

Model outputs including a predictor coding the spectral distance between target and distractor

	Estimate	Std. Error	CI	z	p
		**a) Behavioral Effects**			
Age	-0.53	0.08	[-0.7, -0.37]	-6.42	<.001**
Audibility	0.41	0.01	[0.38, 0.44]	27.77	<.001**
Surprisal	-0.46	0.02	[-0.5, -0.42]	-22.29	<.001**
Audibility:Surprisal	0.17	0.02	[0.14, 0.2]	11.07	<.001**
Word frequency	0.14	0.02	[0.1, 0.19]	6	<.001**
Word entropy	0.21	0.02	[0.17, 0.24]	10.94	<.001**
Audibility:Age	0	0.01	[-0.03, 0.02]	-0.39	.753
Age:Surprisal	0	0.02	[-0.03, 0.03]	0.16	.902
Age:Entropy	0.04	0.02	[0.01, 0.07]	2.8	.009**
		**b) Lower-level TRF fits**			
Acoustic target	0.01	0.01	[-0.01, 0.03]	0.89	.496
Acoustic distractor	0.03	0.01	[0.01, 0.05]	2.44	.023*
Onsets target	0.04	0.01	[0.02, 0.07]	3.34	.002**
Onsets distractor	-0.01	0.01	[-0.03, 0.01]	-0.86	.496
Age:Acoustic target	0	0.01	[-0.03, 0.02]	-0.08	.933
Age:Acoustic distractor	0.01	0.01	[-0.02, 0.03]	0.46	.753
Age:Onsets target	-0.01	0.01	[-0.04, 0.01]	-1.12	.371
Age:Onsets distractor	-0.03	0.01	[-0.06, -0.01]	-2.85	.008**
		**c) Higher-level TRF fits**			
Word target	0.03	0.01	[0.01, 0.06]	2.7	.011*
Age:Word target	0.01	0.01	[-0.02, 0.03]	0.53	.725
		**d) Control variables**			
Pure tone threshold	-0.07	0.02	[-0.1, -0.04]	-4.25	<.001**
Target-to-distractor ratio	0.49	0.01	[0.47, 0.51]	55.89	<.001**
Wordlength	0.08	0.01	[0.07, 0.1]	11.65	<.001**
Word order	-0.13	0.01	[-0.15, -0.11]	-13.36	<.001**
Sentence length	-0.05	0.12	[-0.28, 0.19]	-0.4	.753
Control accuracy	0.24	0.07	[0.1, 0.38]	3.26	.002**
Speech reception threshold	0.29	0.03	[0.23, 0.35]	9.09	<.001**
Spectral distance	-0.02	0.02	[-0.06, 0.02]	-1.11	.371

** p < .01, * p < .05 (FDR corrected)

Minor Concerns

L1; L8–19; L172–186; L210–216; L276–281; L283–290 - Compensation framing exceeds what the current analyses establish.

Issue: The manuscript states that linguistic tracking compensates/offsets age-related distractor effects, but the evidence shown is correlational. Recommendation: Soften wording to reflect correlation, or add a focused analysis that is consistent with compensation (for example an interaction showing that linguistic tracking predicts comprehension more strongly at harder TDRs and/or

when distractor-onset tracking is high). Indicate whether this analysis is confirmatory or exploratory.

Reply: Thank you for this suggestion. In fact, the manuscript includes an additional exploratory analysis focusing on the compensation aspect (Figure 3E and page 13 of the results section). This analysis shows that the positive effect of linguistic tracking on comprehension performance was stronger when distractor onset tracking was high. As stated in the manuscript, this analysis was conducted exploratively. We softened the framing of this result to emphasize its correlational nature.

L. 200-204:

> "Thus, there seems to be a relationship between enhanced distractibility and higher-level tracking. This means that stronger linguistic tracking might compensate for increased individual distractibility. However, based on the available correlational evidence, no causal conclusions on the origin of this relationship can be drawn."

Literature Suggestions

- L49–51 (compensation vs inefficiency/noise interpretation of stronger tracking): add Henry et al. (2017).

- L239–242 (link between higher-level linguistic/semantic tracking and comprehension): add Broderick et al. (2018) and Weissbart et al. (2020).

- L276–281 (mechanistic framing for "compensation" under degraded input): add Rönnberg et al. (2013) and Pichora-Fuller et al. (2016).

- L385–389 (theoretical grounding for surprisal as a predictor): add Levy (2008) and Hale (2001).

- L440–446 (multi-feature TRF methodology / feature separation): add Crosse et al. (2016) and Di Liberto et al. (2015).

Reply: The suggested references were added.

Reviewer #2 (Remarks to the Author):

I co-reviewed this manuscript with one of the reviewers who provided the listed reports. This is part of the Communications Biology initiative to facilitate training in peer review and to provide appropriate recognition for Early Career Researchers who co-review manuscripts.

Reply: Thank you for your constructive feedback which helped us to improve our manuscript.

Reviewer #3 (Remarks to the Author):

This paper highlights key predictors that may contribute to better speech-in-noise listening based on age-related changes. This paper fills gaps in our understanding between attention and speech processing as a function of age. One of the most valuable contributions of this work is including both behavior and speech tracking at the individual sentence level (most related work only has much coarser behavioral information).
The focus of this paper is to understand the age-related compensatory mechanisms in relation to listening to competing speakers. This paper uses EEG, along with behavioral data, to identify which predictors are modulated by compensatory mechanisms and the processing of which speech feature may be affected by age. The paper describes three main results.

Behaviorally, subjects show comprehension benefits from word uncertainty (as measured by lexical entropy) for all ages, but even more so for older adults. Neurally, they first show that comprehension is positively correlated with enhanced representations of the distractor at the acoustic level, and also correlated with enhanced representations of the distractor at the acoustic level, and of the target at the level of phoneme and of the target at the level of phoneme & word onset and phoneme & word statistics/context.

Surprisingly, no overall main effect of age was seen, either behaviorally or neurally. Also, the authors' creative use of graphs to visually explain interaction effects (such as the rightmost graph of Figure 2C) is greatly appreciated. Interaction terms can be very tricky to interpret/explain, and the use of such graphs is very helpful.

Major Comments:

Several of the most critical definitions / use of terminology are very confusing and make it difficult to interpret the results.

Issue 1:

The acoustic predictors include both envelope and envelope onsets, but the term "onset" is widely used throughout the paper and does not refer to acoustic onset predictors.
The term "onset" as most commonly used in the paper applies to word- and phoneme-level onsets. Since words and phoneme use/statistics is language dependent, these are linguistic predictors. The authors do not group them with other linguistic predictors but with the acoustic predictors mentioned above, all together described as "lower-level". This is not problematic by itself, but then that group containing both is often described as containing only non-linguistic predictors, which is both confusing and incorrect.

The group called "higher-level" is entirely made of higher level linguistic predictors, which is fine, but the predictors of this group also referred to as "the linguistic predictors", which is confusing and incorrect since the linguistic onsets describe above are linguistic predictors but are not in this group. Cleaning up these, and related, usages of terminology is most critical.

Reply: We thank the reviewer for pointing out the inconsistent use of terminology. As the reviewer points out, the segmentation of words and phonemes, which is covered by our onsets predictors, requires linguistic processing. We therefore reworded several sections to clarify that the lower-level versus higher-level distinction is not a non-linguistic versus linguistic distinction. Instead of linguistic, we now rely on the terms word-level and probabilistic as descriptors for our higher-level predictors throughout the manuscript, as we believe that these terms provide a more accurate description of the higher-level model. We adjusted the tables and figures accordingly.

Additionally, to avoid the confusion between acoustic onsets and word and phoneme onsets, we replaced the term *acoustic onsets* with the term *envelope derivative*, which is a more concrete reflection of the predictor.

L. 72-77:

> "Investigating speech features at different hierarchically organized levels allowed us to disentangle how lower-level and higher-level linguistic mechanisms support

comprehension performance in a challenging and naturalistic multitalker paradigm. Here, lower-level mechanisms refer to phonetic (i.e., envelope) and phonological (i.e., word and phoneme onsets) processing. Higher-level mechanisms refer to responses to probabilistic features, such as word frequency, word surprisal, and word entropy."

L. 161-164:

"Although the tracking of distractor acoustics was positively related to comprehension, we observed a negative effect of distractor onsets in older adults. This might indicate that, while acoustic analysis is required for comprehension, tracking of the intermediate onset level marks a distraction effect that is detrimental for comprehension."

L. 166-170:

"To investigate the neural tracking of higher-level features, we conducted a separate TRF analysis predicting the residualized EEG responses from word-level (word frequency, surprisal, and entropy) and phoneme-level (phoneme surprisal and phoneme entropy) probabilistic features.

As displayed in Figure 3A, the word level probabilistic features were significantly tracked for the target stream. "

L. 178-179:

"As displayed in Figure 3 B, there was a positive effect of age on higher-level target tracking at the word level."

L. 256-258:

"We investigated neural tracking for the target and distractor streams across several levels of the speech hierarchy, from phonetic and phonological (lower-level) mechanisms to higher-level probabilistic features."

L. 289-294:

"A positive correlation between age and the strength of word-level encoding for the target stream indicates that the neural tracking of higher-level probabilistic features was stronger in older adults than in younger adults. Using a time-resolved measure of encoding accuracy, we found that, when word-level probabilistic features could reliably predict brain activity (in a late processing window starting at 400 ms after word onset), a positive association between age and word-level encoding performance emerged."

L. 300-304:

"This is supported by our evidence showing that increased word-level tracking can compensate for detrimental effects of a stronger distractor representation across

the adult life span. Thus, an increased reliance on higher-level processing may serve as a strategy to maintain successful speech comprehension in challenging listening conditions across the adult life span.”

Figure 3: Neural tracking and neuro-behavioral correlations for the higher-level target features

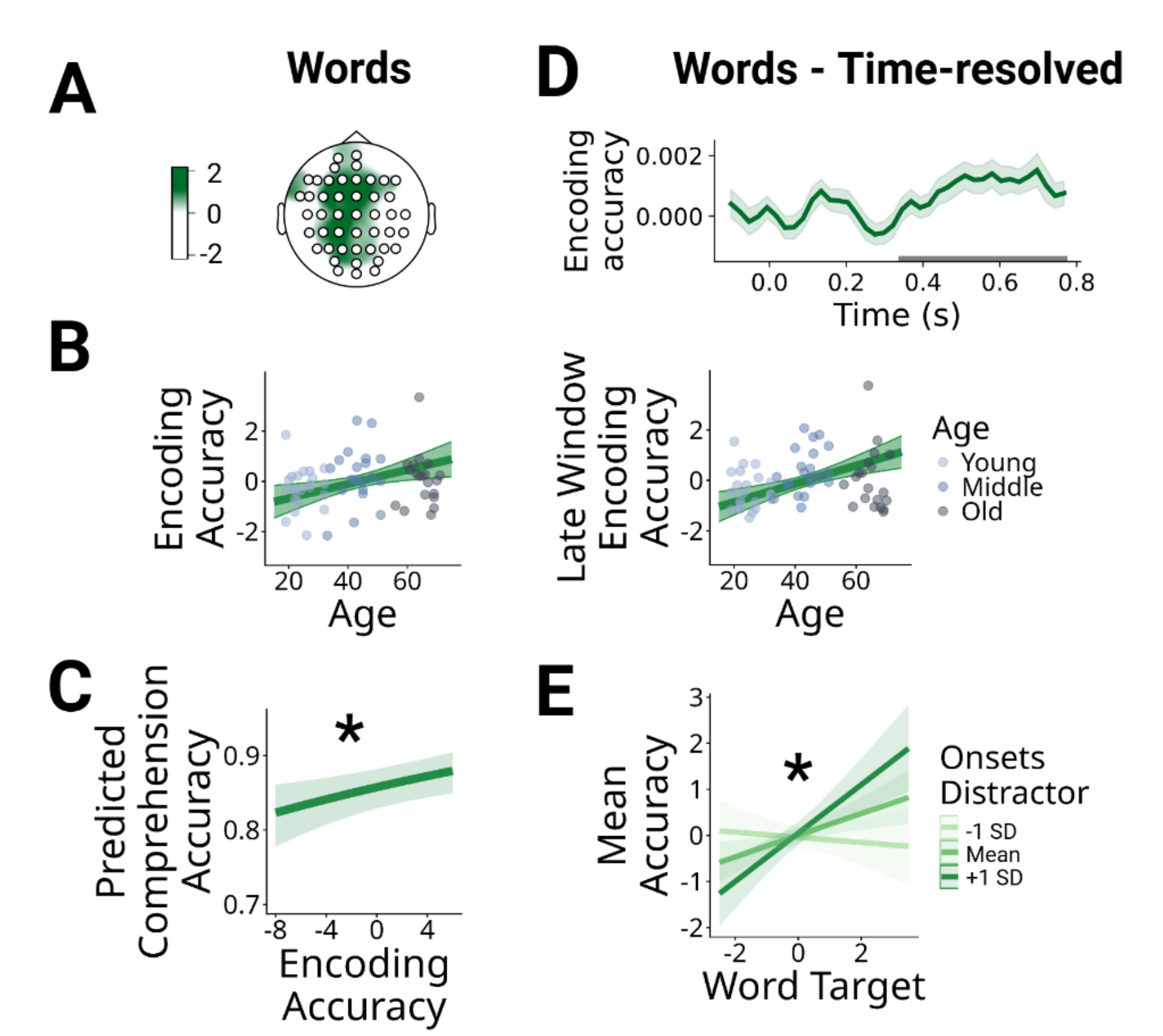

A: Topographies for the encoding accuracies. Significant electrodes in mass-univariate t-tests are marked by white dots. B: Age effects on encoding accuracy. Significant effects are marked by regression lines. There was a positive correlation between age and word-level encoding accuracy. C: Neuro-behavioral correlations. Linear mixed model predictions of the effects of trial-wise neural encoding accuracy on comprehension accuracy. Positive effects of word-level target tracking on comprehension accuracy. D: Time-resolved encoding accuracy for the target stream and age effect on encoding accuracy in a late window (400 - 800 ms). Grey bars indicate significant encoding performance. E: Word-level tracking may compensate for increased distraction. Significant interaction between the tracking of word-level target features and distractor onsets (Estimate = 0.03, SE = 0.01, t = 2.66, p = .023*). The positive effect of word-level target tracking on comprehension accuracy was larger at high levels of distractor onsets tracking, indicating a potential compensatory function of higher-level tracking.

Table 2: Feature groups

Model variant	Shuffled features
Acoustic	Envelope, envelope derivative
Onsets	Word onsets, phoneme onsets
Phoneme level	Phoneme surprisal, phoneme entropy
Word level	Word surprisal, word frequency, word entropy

Table 1: Model outputs predicting comprehension performance

	Estimate	Std. Error	CI	z	p
**a) Behavioral effects**					
Age	-0.53	0.08	[-0.7, -0.37]	-6.43	<.001**
Audibility	0.41	0.01	[0.38, 0.44]	27.77	<.001**
Surprisal	-0.46	0.02	[-0.5, -0.42]	-22.29	<.001**
Audibility:Surprisal	0.17	0.02	[0.14, 0.2]	11.07	<.001**
Word frequency	0.15	0.02	[0.1, 0.19]	6.01	<.001**
Word entropy	0.21	0.02	[0.17, 0.24]	10.95	<.001**
Audibility:Age	0	0.01	[-0.03, 0.02]	-0.39	.756
Age:Surprisal	0	0.02	[-0.03, 0.03]	0.16	.905
Age:Entropy	0.04	0.02	[0.01, 0.07]	2.8	.009**
**b) Lower-level TRF fits**					
Acoustic target	0.01	0.01	[-0.01, 0.03]	0.89	.478
Acoustic distractor	0.03	0.01	[0.01, 0.05]	2.45	.022*
Onsets target	0.04	0.01	[0.02, 0.07]	3.35	.002**
Onsets distractor	-0.01	0.01	[-0.03, 0.01]	-0.86	.478
Age:Acoustic target	0	0.01	[-0.03, 0.02]	-0.08	.933
Age:Acoustic distractor	0.01	0.01	[-0.02, 0.03]	0.46	.730
Age:Onsets target	-0.01	0.01	[-0.04, 0.01]	-1.13	.370
Age:Onsets distractor	-0.03	0.01	[-0.06, -0.01]	-2.85	.008**
**c) Higher-level TRF fits**					
Word target	0.03	0.01	[0.01, 0.06]	2.7	.011*
Age:Word target	0.01	0.01	[-0.02, 0.03]	0.53	.698
**d) Control variables**					
Pure tone threshold	-0.07	0.02	[-0.1, -0.04]	-4.25	<.001**
Target-to-distractor Ratio	0.49	0.01	[0.47, 0.51]	55.89	<.001**
Wordlength	0.08	0.01	[0.07, 0.1]	11.64	<.001**
Word order	-0.13	0.01	[-0.15, -0.11]	-13.35	<.001**
Sentence length	-0.1	0.11	[-0.32, 0.12]	-0.9	.478
Control accuracy	0.24	0.07	[0.1, 0.38]	3.26	.002**
Speech Reception threshold	0.29	0.03	[0.23, 0.35]	9.11	<.001**

** p < .01, * p < .05 (FDR corrected)

Issue 2:

Task difficulty was individually adjusted based on the individual speech reception threshold (SRT) to control for effects of peripheral constraints. This is an important component of the experiment, but almost no information is given regrading how this was applied to the subjects.

The individual task difficulty was adjusted based on an adaptive staircase measure to ensure that any observed age effects rely on a differential use of strategies rather than different task difficulties due to peripheral hearing differences. We clarified this in the introduction.

L. 62-66:

> "Additionally, task difficulty was individually adjusted based on the individual speech reception threshold (SRT) as measured by an adaptive staircase procedure to control for effects of peripheral constraints. As a result, the average comprehension performance was balanced across participants to allow for conclusions regarding the use of different strategies to achieve similar performance levels."

In particular, given the surprising finding that no overall main effect of age was seen for comprehension, is it possible that the expected main age effect was negated by customizing SRT? If so, this is important to know, but impossible for the reader to see given the lack of SRT results (especially SRT x age). Even if not, addressing the lack of overall main effect of age would be important regardless.

Reply: The lack of an age effect in the mixed effects model can be attributed to the control for other related variables, especially the pure tone average (PTA). As age and the PTA are highly correlated (r = .78), it is difficult to attribute any explained variance to one of the two variables. In the subject-level models, we therefore opted to residualize age from the PTA before adding it into the model as an additional variable. As a consequence, only the variance in hearing ability that cannot be explained by age will be entered into the model as a covariate. In the original version of the manuscript, this was not done for the main mixed effects model, but instead the non-adjusted version of the PTA was added as a control variable. We believe that it is the more appropriate approach to unify this in the full manuscript. We clarified this in the methods.

As a result of adding the residualized PTA to the model, the analysis now reveals the negative effect of age on comprehension performance, which was expected by the reviewer. We adjusted this in the introduction and the results sections. No other effects

changed as a result of adjusting the predictor variable. The adjusted model outputs can be retrieved from our response to the reviewer's first issue.

L. 540-542:

> "The analysis was controlled for word frequency, word length, sentence length (in words), TDR condition, SRT, orthogonalized PTA, and control accuracy by adding these variables as additional predictors."

L. 101-102:

> "Overall, there were negative effects of age and PTA on comprehension performance."

In a related context, regarding Controlling for Correlated Variables (Page 11, Line 178/179; Page 24, Line 476; Page 25, Line 494): How was the analysis controlled for these variables (including SRT)? It looks like the only thing described is PTA (Line 478).

Reply: Generally, we controlled for the correlated variables by adding them as additional predictors into our linear mixed model analyses. Only in the case of the PTA, we applied a different procedure due to its strong correlation with age. We clarified this in the methods section.

L. 518-519:

> "The analysis was controlled for the SRT and the mean accuracy in the main experiment and in the control condition by adding these variables as additional predictors."

L. 540-542:

> "The analysis was controlled for word frequency, word length, sentence length (in words), TDR condition, SRT, PTA, and control accuracy by adding these variables as additional predictors."

Issue 3:

The audibility measure is never defined. Is it the same thing as the amount of available glimpses? Regardless such a critical measure should be carefully defined.

Reply: We thank the reviewer for spotting this oversight and we added details on the computation of the glimpse rate in the methods section.

L. 534-537:

> "The glimpse rate relies on a comparison between the spectrogram amplitudes of the target and distractor streams. At each target word segmentation, we defined the proportion of spectrotemporal events that exceed a local target-to-distractor threshold. Based on previous work, this local threshold was set to -5 dB."

Lower-Level Comments:

Issue 4:

Page 5, Lines 96-99; Page 6, Figure 1C; Page 14, Line 224, and others

"…word entropy had a stronger positive effect on comprehension accuracy." Would the authors please expand on this phenomenon, explanation and interpretation? It seems counterintuitive that, regardless of age, greater uncertainty leads to better predicted accuracy. Addressing this confusion (even in the results section) would benefit the reader. Additionally, it seems that figure 1C could also be alternatively interpreted as that word certainty has a stronger negative effect on older adults (which seems like a more direct interpretation), and more guidance as to why the reader should not take that interpretation would be helpful.

Reply: We agree that the positive effect of entropy on comprehension performance seems unintuitive. In fact, several previous studies have also revealed positive effects of entropy on comprehension or processing efficiency. We added the corresponding references and clarified the interpretation in the results section.

Concerning the age effect, we now explicitly mention the additional interpretation provided by the reviewer.

L. 98-101:

> "While this effect seems counterintuitive, it is consistent with previous studies showing more efficient lexical processing in contexts of high uncertainty [43,44]. This effect could indicate that the allocation of attention as well as the pre-activation of semantic features is increased in contexts of high uncertainty."

L. 107-108:

> "More precisely, there seems to be a stronger negative effect of low word entropy on comprehension performance with increasing age."

Issue 5:

TRF Calculation: It seems that two separate mTRF sets were calculated, one for the lower-level predictors and one for the higher-level predictors (using the residuals of the first set). Why were they not all calculated as an mTRF set that combines all acoustic and linguistic predictors simultaneously? Using the two stage method runs the risk of removing responses due to higher-level that have some correlation with lower level predictors.

Reply: Our approach of residualizing lower-level features from the higher-level analysis was motivated by the need to disentangle neural responses to higher-level linguistic features from those driven by lower-level acoustic structure. As the reviewer notes, acoustic and linguistic predictors are often substantially correlated. When included simultaneously in a single mTRF model, this collinearity can make it difficult to attribute variance uniquely to higher-level representations. Our approach ensures that the higher-level model captures variance that cannot be explained by acoustic structure alone, thereby providing a more conservative estimate of higher-level tracking.

We agree that the two-stage method including the residualization procedure runs the risk of over-penalizing the higher-level analysis. However, given the strong collinearity between the acoustic and the linguistic predictors, we believe that residualization is the more conservative and clean method to isolate the unique contributions of higher-level processing.

We extended the explanation in the methods section to clarify this rationale.

L. 475-477:

> "This was important to isolate the unique contributions of the higher-level features, given the high collinearity between the lower-level and the higher-level features in natural speech."

Issue 6:

Multicolinearity: Page 25, Line 494, Line 497-498

"The analysis was controlled for word frequency, sentence length (in words), ... We included interactions between age and all variables of interest (word surprisal, entropy, and audibility, TRF model fits)." This goes back to the previous comment about how the analysis was controlled for these variables. In Table 1, word frequency is used as a fixed effect, but it is not mentioned in the variables of interest. If word frequency should be included in the variables of interest, it is surprising that there were no issues with multicollinearity, as some issues between word surprisal and word frequency would be expected.

Reply: As clarified above, controlling for a variable means that this variable was included as an additional fixed effect in the linear mixed model. Therefore, it is correct that word frequency was included as a fixed effect, although it was not a primary variable of interest. The correlation between word surprisal and word frequency in our stimulus set was r = -.67. Thus, 45% of the variance in word surprisal can be explained by word frequency. Although being highly significant, the correlation still seems to leave enough individual variance to each of the variables, which is reflected in their moderate variance inflation factors. We clarified this in the methods section.

L. 551-553:

> "The highest variance inflation factors were observed for word surprisal and word frequency. However, despite their substantial correlation (r = -.67), we assume that there was sufficient non-redundant information in both predictor variables."

Issue 7:

Page 11, Line 179 to Page 13, Line 183.

"Results revealed a significant interaction between the linguistic target and the onsets distractor encoding accuracies. This interaction indicates that the positive effect of linguistic target tracking on comprehension performance is stronger when the distractor onset tracking is increased (see Figure 3E). This indicates that stronger linguistic tracking might compensate for increased individual distractibility."

This exploratory analysis was very interesting. However, since the model only looked at target linguistic tracking and distractor onset tracking, it is unclear whether this is the compensatory mechanism or if individuals who have an overall higher tracking of all predictors have better performance. One might add an additional variable in the model, such as distractor acoustic tracking, to verify that the compensatory mechanism is indeed the combination of these two variables rather than overall higher tracking.

Reply: In fact, the linear mixed model already included distractor onset tracking as well as distractor acoustic tracking to investigate the specificity of the observed interaction. We clarified this in the results section and included the full model output table in the supplementary material.

L. 190-192:

> "We used a multiple regression model predicting each subjects' mean accuracy from the mean encoding accuracy of the word-level target features and the tracking of the distractor onsets and acoustics and their interactions."

L. 199-200:

> "The effect was specific for the neural tracking of the distractor onsets, with no significant interaction emerging for distractor acoustic tracking."

Table S1: Model Outputs for the Compensation Analysis

	Estimate	Std.Error	CI	t	p
Age	-0.66	0.09	[-0.84, -0.48]	-7.28	<0.001**
Word Target	0.23	0.08	[0.07, 0.39]	2.94	.011*
Onsets Distractor	0.05	0.09	[-0.13, 0.22]	0.51	.707
Acoustic Distractor	0.07	0.09	[-0.11, 0.25]	0.75	.624
Word Target*Onsets Distractor	0.29	0.09	[0.1, 0.48]	3.09	.008**
Word Target*Acoustic Distractor	-0.18	0.12	[-0.43, 0.06]	-1.48	.224
Age*Word Target	-0.01	0.09	[-0.2, 0.17]	-0.13	.901
Age*Onsets Distractor	-0.17	0.08	[-0.34, 0]	-2.01	.093
Age*Acoustic Distractor	0.14	0.09	[-0.05, 0.32]	1.46	.224
Age*Word Target*Onsets Distractor	-0.04	0.1	[-0.24, 0.17]	-0.37	.763
Age*Word Target*Acoustic Distractor	0.07	0.12	[-0.17, 0.32]	0.61	.681
Control Accuracy	0.27	0.08	[0.11, 0.43]	3.36	.005**
PTA (residualized)	-0.41	0.09	[-0.59, -0.23]	-4.62	<0.001**
SRT	0.3	0.04	[0.23, 0.37]	8.36	<0.001**

** $p < .01$, * $p < .05$ (FDR corrected)

Issue 8:

Page 23, Line 445-448

"The regularization parameter was fitted separately for acoustic and linguistic models. The optimal regularization parameter was determined based on the median across participants, as the mean regularization was strongly driven by a small number of outliers displaying very high regularization parameters."

Would you please expand why you used the same regularization parameter across participants? There are benefits to this (which would be good to hear expressed), but

there is also the potential for concern that the same regularization parameter across such a large age range might bias the results in some way: e.g., if older adults have noisier baselines than younger adults. Were the subjects with very high regularization parameters the older adults?

Reply: Fitting one regularization parameter per participant led to clear signs of overfitting in the acoustic models, probably because of the limited amount of available data. We therefore opted to fit the regularization parameters across participants. There were no significant correlations between age and the individual regularization parameters ($r = -0.15$, $p = .22$ for the lower-level model and $r = -0.04$, $p = .78$) for the higher-level model. We therefore assume that the choice of fitting common regularization parameters did not impact the age effects. We added this information to the manuscript.

L. 482-484:

> "Fitting individual regularization parameters per participant led to of overfitting in the acoustic analyses and we therefore opted to fit the regularization parameters across participants."

L. 487-488:

> "There was no correlation between age and the individual regularization parameters, suggesting that the model fitting procedure did not bias the age effects."

Minor Comments:

Page 9, Figure 2C

The positive slope of the acoustic distractor seems at odds with the non-positive slopes of the onsets distractor. This is explained in the discussion but an acknowledgment of this and/or a quick explanation in results would greatly benefit the reader trying to parse the large number of results.

Reply: We clarified this distinction in the results section.

L. 145-147:

> "This could indicate that an initial acoustic analysis of the distractor stream and segmentation of the target stream are beneficial for stream segregation and comprehension performance."

L. 161-164:

> "Although the tracking of distractor acoustics was positively related to comprehension, we observed a negative effect of distractor onsets in older adults. This might indicate that, while acoustic analysis is required for comprehension,

tracking of the intermediate onset level marks a distraction effect that is detrimental for comprehension."

Page 14, Line 224-228

"[The positive effect of word entropy on comprehension performance] got stronger with increasing age, ... In line with this conclusion, older adults seemed to benefit more from overall sentence context, when comparing comprehension performance in sentences and word lists."

- **It is not clear how the positive effect of word entropy is "in line with the conclusion" that older adults benefit from sentence context. These seem like opposite statements. Would you please explain?**

Reply: We clarified this statement in the discussion section.

L. 247-250:

> "This means that the allocation of attention in response to highly uncertain contexts, as well as the deallocation of attention in contexts deemed rather certain seems to be stronger in older adults. Thus, the context certainty seems to be a stronger predictor of older adults' comprehension performance."

Page 21, Line 406/407

"All features were min-max normalized to avoid negative values before entering the TRF."

- **Why are negative values avoided? It is very common in TRF analysis to z-score predictors (and responses), which allows negative values. Also, min-max normalization is susceptible to random scaling artifacts due to min/max outliers (which could be avoided by, e.g., z-scoring or 5/95 percentile normalization).**

Reply: We chose not to z-score the predictors because many of our features consist of sparse event sequences (i.e., long stretches of zeros with occasional non-zero entries). Z-scoring such variables would shift the baseline away from zero and introduce negative values. Preserving this meaningful zero baseline was important for interpretability, particularly for binary features such as word and phoneme onsets. We clarified this reasoning in the methods section.

L. 433-434:

> "All features were min-max normalized to retain the natural zero baseline for the sparse regressors before entering the TRF."

Scaling for Figures

- **Some variables look like they have been z-scored (audibility in Figure 1B), but this is not explained in the methods. If so, would you please add this to the methods?**

Reply: We clarified this in the methods.

L. 546-548:

> "All variables were z-scored before entering the regression model. With regards to age, the original age values are used only for illustration purposes."

"Trial-wise TRFs"

- **It is unclear what is meant by trial-wise TRFs. Based on the methods, it seems like a single TRF is created (page 22), but "trial-wise" TRFs sounds like one TRF is created for each trial.**

Reply: We clarified this in the methods section. Throughout the manuscript, we made sure to refer to trial-wise TRF model fits or encoding accuracy instead of trial-wise TRFs.

L. 466-467:

> "We obtained trial-wise model fits for each individual trial from the TRF models trained across trials."

L. 66-67:

> "Using multivariate temporal response function (TRF) modeling, we provide insight into the trial-wise neural tracking strength of acoustic and linguistic speech representations (...)"

Page 21, Line 409

"As mentioned above, target and distractor sentences were presented by a male and a female voice, respectively."

- **It is unclear whether there are is a single female and single male speaker across all trials or if there are different female/male speakers.**

Reply: We clarified this in the methods section.

L. 437:

> "There was only one male and one female speaker across the full experiment."

Page 8, Table 1

- **Should the interaction terms in the table have asterisks or colons? Based on the notation of R, an asterisk represents not only the interaction of the variables, but also includes the main effect; a colon would only represent the interaction itself.**

Reply: We adjusted the table to match the default notation in R. The updated model table can be retrieved from our response to the reviewer's first issue.

Page 9, Figure 2 (and other locations, e.g. Page 11, Line 170)

"No significant effects of age on encoding accuracy strength were observed."

- **Please change this to "main effect of age" to avoid confusion regarding the presence of interaction terms with age.**

Reply: This was added.

Pages 9-10, Lines 153-154

"As displayed in Figure 3 A, the higher-level linguistic features were only significantly tracked for the target stream."

- **Figure 3A does not show this (i.e., it does not show that the higher-level linguistic features were not significantly tracked for the distractor stream).**

Reply: We removed the word "only" to emphasize the significant tracking of the target stream.

Page 20, Line 389

- **"N" is not defined**
- **This equation has an equation label as (1) but the other equations do not**

Reply: This was fixed and more explanation was provided to explain the equation.

L. 414-415:

> "Word entropy is defined as the uncertainty of the upcoming word given the previous sentence context from the previous word w_{i-1} to the first word in the sentence w_{i-n}."

Word TRF

- **Currently, the "word TRF" is a combination of word entropy, surprisal, and frequency. Because there were behavioral results that saw an age effect with word entropy, we are curious to see what the effect of age is on the word entropy TRF.**

Reply: We did not observe an effect of age on the TRF weights for entropy. Below, we provide a figure containing the standardized TRF weights for word entropy divided by three age groups obtained by segmenting our sample into three equally sized age groups for the reviewer's inspection. We performed cluster-level permutation tests using threshold-free cluster enhancement separately for each group and found that the TRF only significantly differed from zero for the oldest group. However, when comparing the groups to each other using cluster-wise permutation tests, no significant differences emerged. We therefore retain from interpreting any age-related differences in the entropy TRF weights.

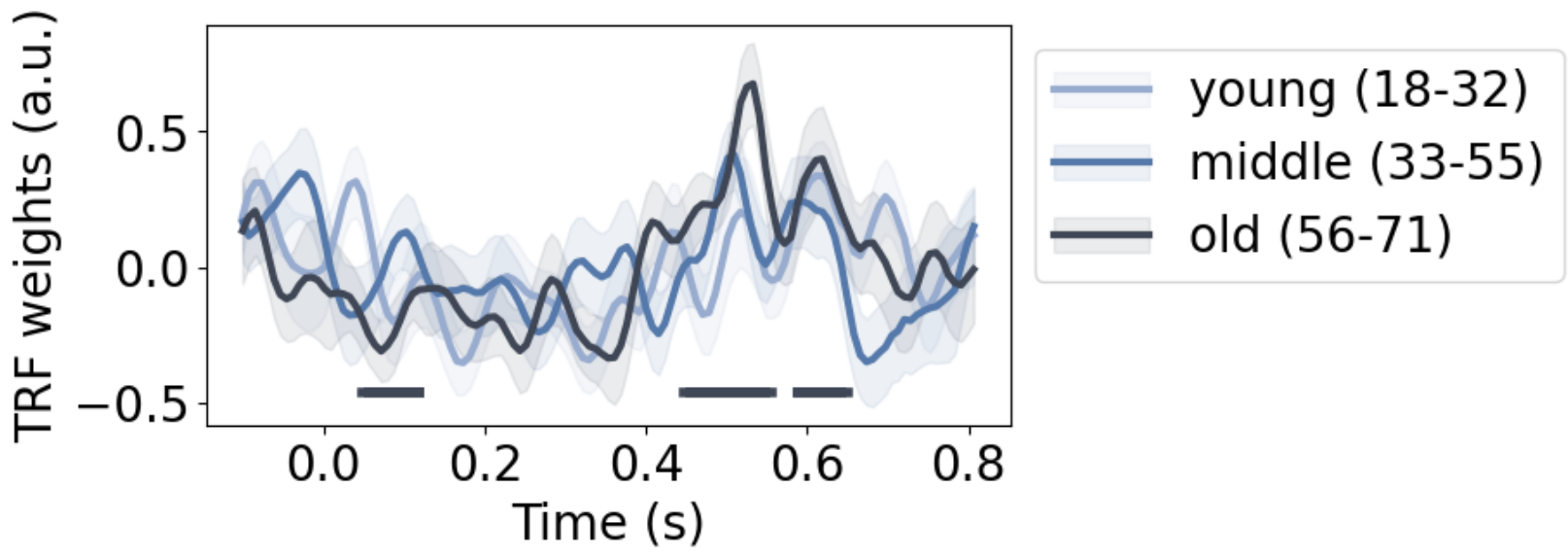

Page 12, Figure 3D

- **You might change the y-axis title to include that it is the late window encoding accuracy, to avoid confusion from Figure 3B.**

Reply: This was adjusted.

Page 16, Lines 269-270

"This late processing window is typically associated with the processing of expectations and word identity "

- **Please consider citing Ref. 44 here also, regarding the role of the late processing window and expectations and word identity; it is a more closely related case than the currently cited papers since the TRF framework was used there (like here).**

Reply: We added the reference.

Typos

Page 6, Figure A caption first line

- **“by” → “of”?**

Page 18, Line 322

- **“latter” → “last”?**

Page 24, Line 485

- **capitalize Whisper**

Page 24, Line 486

- **possible typo, “controlled”**

Reply: The typos were fixed.

Manuscript COMMSBIO-26-1021

Response to Reviewers

Dear Editors,

We would like to thank you for the letter dated June 23, and the opportunity to submit a revised copy of the manuscript “Linguistic information may compensate for age-related decline in attentional filtering” for consideration at Communications Biology.

We would like to thank the reviewers for their time and effort, their positive evaluation, and their constructive feedback. We comprehensively revised the manuscript along the following main points:

- We adjusted the wording in the title, abstract, and methods sections
- We removed the speaker feature control
- We clarified sections of Table 1 and Figure 3

We believe that these changes have resulted in an improved revised manuscript, which you will find uploaded alongside this document. We hope that our manuscript may ultimately be deemed suitable for Communications Biology. Additionally, we provide detailed point-by-point responses to the reviewers’ comments below. Reviewer comments are provided in bold font.

Point-by-Point Response

Reviewer #1 (Remarks to the Author):

We thank the authors for their detailed response and for the revisions to the manuscript. Our assessment focuses on the points raised in our previous report.

Reply: We thank the reviewer for their positive assessment of our manuscript and their constructive feedback. We hope that we have addressed all concerns as detailed below.

The main methodological concerns from our report have largely been addressed. The trial-wise TRF model-fit measure is now described more clearly, and the added 10-fold cross-validation analysis with independent test folds provides a useful stability check. The residualization concern has also been addressed by rerunning the linguistic TRF analysis with cross-validated acoustic residualization. The removal of the phoneme-level linguistic fits after they no longer survived this procedure is appropriate.

The TRF inference procedure is now better specified, with increased shuffle iterations and added details on TFCE, permutations, sensor adjacency, and correction scope.

Issue 1:

The authors have also responded adequately to the stimulus- and design-related concerns. They now report the proportion of GPT-generated sentences, provide the prompts, and include a stimulus-source control model. The source effect suggests that GPT-generated sentences were easier to understand, but the main results appear stable after including this covariate; this should be reported transparently.

Reply: We now report the results of the control analysis when including the covariate in the supplementary material to improve transparency. Additionally, we reference this supplementary table in the main manuscript.

l. 317:

> *"All results remained stable after adding a control predictor for the sentence origin (corpus versus GPT, see Table S3)."*

Table S3: Model outputs including a predictor coding the sentence origin

	Estimate	Std. Error	CI	z	p
**Behavioral Effects**					
Age	-0.53	0.08	[-0.7, -0.37]	-6.42	<.001**
Audibility	0.41	0.01	[0.38, 0.44]	27.81	<.001**
Surprisal	-0.46	0.02	[-0.5, -0.42]	-22.23	<.001**
Audibility:Surprisal	0.17	0.02	[0.14, 0.2]	11.06	<.001**
Word frequency	0.15	0.02	[0.1, 0.19]	6.07	<.001**
Word entropy	0.21	0.02	[0.17, 0.24]	10.94	<.001**
Audibility:Age	0	0.01	[-0.03, 0.02]	-0.39	.783
Age:Surprisal	0	0.02	[-0.03, 0.03]	0.17	.898
Age:Entropy	0.04	0.02	[0.01, 0.07]	2.79	.009**
**Lower-level TRF fits**					
Acoustic target	0.01	0.01	[-0.01, 0.03]	0.89	.460
Acoustic distractor	0.03	0.01	[0.01, 0.05]	2.44	.023*
Onsets target	0.04	0.01	[0.02, 0.07]	3.33	.002**
Onsets distractor	-0.01	0.01	[-0.03, 0.01]	-0.88	.460
Age:Acoustic target	0	0.01	[-0.03, 0.02]	-0.09	.927
Age:Acoustic distractor	0.01	0.01	[-0.02, 0.03]	0.45	.765
Age:Onsets target	-0.01	0.01	[-0.04, 0.01]	-1.11	.357
Age:Onsets distractor	-0.03	0.01	[-0.06, -0.01]	-2.85	.008**
**Higher-level TRF fits**					
Word target	0.03	0.01	[0.01, 0.05]	2.4	.024*
Age:Word target	0	0.01	[-0.02, 0.03]	0.3	.819
**Control Variables**					
Pure tone threshold	-0.33	0.08	[-0.49, -0.18]	-4.25	<.001**
Target-to-distractor ratio	0.49	0.01	[0.47, 0.51]	55.91	<.001**
Wordlength	0.23	0.02	[0.19, 0.27]	11.65	<.001**
Word order	-0.13	0.01	[-0.15, -0.11]	-13.31	<.001**
Sentence length	-0.12	0.08	[-0.28, 0.05]	-1.4	.226
Control accuracy	0.24	0.07	[0.1, 0.38]	3.26	.002**
Speech reception threshold	0.29	0.03	[0.23, 0.35]	9.1	<.001**
Source [GPT]	0.65	0.17	[0.32, 0.97]	3.88	<.001**

** p < .01, * p < .05 (FDR corrected)

The fixed speaker-role mapping remains a design limitation, but the added spectral-distance control is a reasonable post hoc check, and the rationale for not counterbalancing speaker roles is now clearer.

The reporting of the German GPT-2 surprisal and entropy predictors has improved. The Methods now specify the model, tokenizer, context definition, and token-to-

word aggregation procedure. The authors did not run an alternative language-model sensitivity analysis, explaining that they relied on a pre-trained model and did not have access to alternative checkpoints. This does not fully address the sensitivity point, but we do not consider it a major remaining obstacle if the predictor computation is reproducible from the shared code.

Issue 2:

One issue remains at the level of framing. The Results section now includes a clearer caveat that the compensation analysis is correlational and exploratory. However, the title and abstract still use stronger language than the evidence warrants, including "compensates," "direct evidence," "directly show," and "offset." We recommend softening the title and abstract to match the correlational nature of the evidence. Phrasing such as "is associated with," "may support," or "is consistent with a compensatory role" would better reflect the inferential strength of the analyses.

Reply: We adjusted the wording in the title and abstract to soften the conclusions, as suggested by the reviewer.

Title: Linguistic information may compensate for age-related decline in attentional filtering

l. 8:

> *"Combining trial-resolved multivariate temporal response function modeling with detailed behavioral comprehension responses, we provide a window into lower-level impairments and possible higher-level compensatory mechanisms across the adult life span. Neuro-behavioral correlations on a trial-by-trial level show that reduced behavioral performance in late adulthood is associated with increased distractor representation."*

l. 18:

> *"Specifically, we show that increased reliance on higher-level processing could attenuate age-related impairments in attentional filtering."*

Issue 3:

We also recommend one small wording correction in the Methods. The manuscript describes accuracy in the control condition as "serving as a proxy for working memory performance." This measure is better described as a proxy for general task performance, attention, or performance under highly intelligible listening conditions, rather than working memory specifically.

Reply: We replaced “working memory” by “performance under highly intelligible listening conditions”, as suggested by the reviewer.

l. 569:

> *“The analysis was controlled for the SRT, orthogonalized PTA and the accuracy in the control condition, serving as a proxy for performance under highly intelligible listening conditions, likely driven by working memory.”*

In summary, the authors have addressed our main technical concerns. We do not see a need for another major revision from our side. The remaining changes are targeted wording revisions: calibrating the compensation language in the title and abstract, and revising the description of control-condition accuracy. Once these changes are made, we would consider the manuscript responsive to our previous review.

Reviewer #2 (Remarks to the Author):

I co-reviewed this manuscript with one of the reviewers who provided the listed reports. This is part of the Communications Biology initiative to facilitate training in peer review and to provide appropriate recognition for Early Career Researchers who co-review manuscripts.

Reply: We thank the reviewer for their positive assessment of our manuscript and their constructive feedback. We hope that we have addressed all concerns as detailed below.

Reviewer #3 (Remarks to the Author):

Thank you for your responsiveness to our review.

Only a few minor issues remain.

Reply: We thank the reviewer for their positive assessment of our manuscript and their constructive feedback. We hope that we have addressed all concerns as detailed below.

Page 8, Table 1.

Please consider adding the control variables for each model within this table, which might be helpful for the reader.

Reply: Table 1 presents the results of only one regression model predicting behavioral comprehension performance from the speech variables, neural tracking estimates, as well as the control variables. Therefore, the listed control variables refer to the full model.

We clarified this by adding this information in the table legend, removing the letters a to d within the table, and adding this information in the methods section. We append the updated table below.

Table 1: Model outputs predicting comprehension performance

	Estimate	Std. Error	CI	z	p
		**a) Behavioral effects**			
Age	-0.53	0.08	[-0.7, -0.37]	-6.42	<.001**
Audibility	0.41	0.01	[0.38, 0.44]	27.77	<.001**
Surprisal	-0.46	0.02	[-0.5, -0.42]	-22.29	<.001**
Audibility:Surprisal	0.17	0.02	[0.14, 0.2]	11.07	<.001**
Word frequency	0.15	0.02	[0.1, 0.19]	6.01	<.001**
Word entropy	0.21	0.02	[0.17, 0.24]	10.95	<.001**
Audibility:Age	0	0.01	[-0.03, 0.02]	-0.39	.787
Age:Surprisal	0	0.02	[-0.03, 0.03]	0.17	.900
Age:Entropy	0.04	0.02	[0.01, 0.07]	2.79	.009**
		**b) Lower-level TRF fits**			
Acoustic target	0.01	0.01	[-0.01, 0.03]	0.89	.472
Acoustic distractor	0.03	0.01	[0.01, 0.05]	2.44	.023*
Onsets target	0.04	0.01	[0.02, 0.07]	3.33	.002**
Onsets distractor	-0.01	0.01	[-0.03, 0.01]	-0.87	.472
Age:Acoustic target	0	0.01	[-0.03, 0.02]	-0.09	.929
Age:Acoustic distractor	0.01	0.01	[-0.02, 0.03]	0.44	.773
Age:Onsets target	-0.01	0.01	[-0.04, 0.01]	-1.1	.385
Age:Onsets distractor	-0.03	0.01	[-0.06, -0.01]	-2.85	.008**
		**c) Higher-level TRF fits**			
Linguistic word target	0.03	0.01	[0.01, 0.05]	2.39	.025*
Age:Linguistic word target	0	0.01	[-0.02, 0.03]	0.31	.820
		**d) Control variables**			
Pure tone threshold	-0.33	0.08	[-0.49, -0.18]	-4.25	<.001**
Target-to-distractor Ratio	0.49	0.01	[0.47, 0.51]	55.9	<.001**
Wordlength	0.23	0.02	[0.19, 0.27]	11.64	<.001**
Word order	-0.13	0.01	[-0.15, -0.11]	-13.35	<.001**
Sentence length	-0.08	0.08	[-0.24, 0.09]	-0.91	.472
Control accuracy	0.24	0.07	[0.1, 0.38]	3.26	.002**
Speech Reception threshold	0.29	0.03	[0.23, 0.35]	9.1	<.001**

Results from one logistic mixed effects model predicting the behavioral comprehension accuracy from speech features, trial-wise neural tracking estimates, and control variables.
** $p < .01$, * $p < .05$ (FDR corrected)

l. 543:

> *"Only one mixed effects model was used to investigate the effects of the speech features and the neural tracking estimates on behavioral comprehension performance."*

Page 12, Figure 3, Panel E.

Please change “Mean Accuracy” on y-axis to “Mean Comprehension Accuracy” to avoid confusion with encoding accuracy, and to better align with the text description in the figure caption.

Reply: Thank you for this suggestion, we adjusted the y-axis label accordingly.

Figure 3: Neural tracking and neuro-behavioral correlations for the higher-level target features

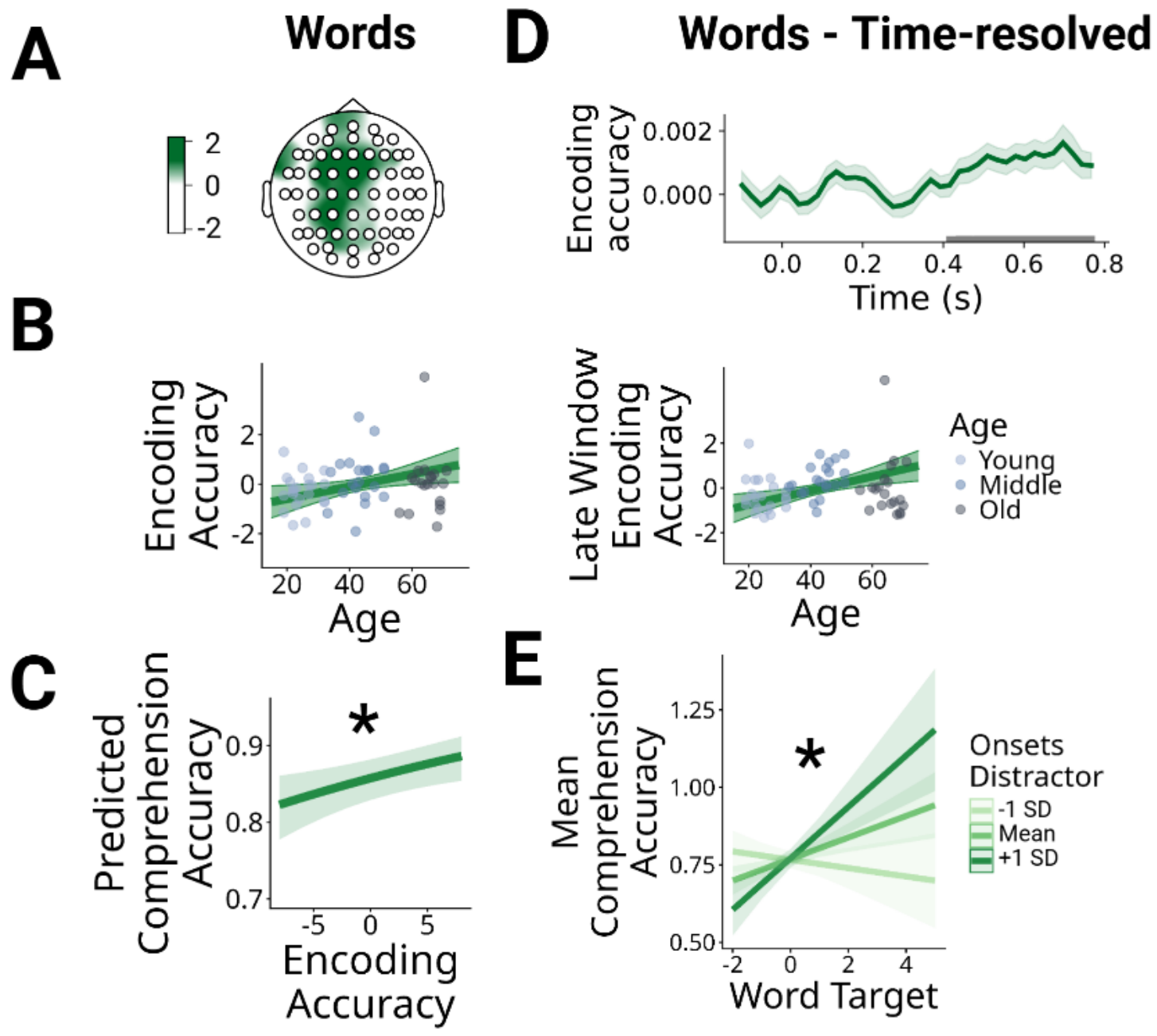

Page 23, Lines 450-451, “Speaker characteristics”.

There are only 6 characteristics listed here, not 7.

Additionally, since there is only one male and one female speaker in this experiment, I’m not sure what the purpose is for including these characteristics at each word onset. Was this a separate predictor from word onset, or was each word onset multiplied by the mean rating before creating the acoustic model? If the former, I worry about the stimulus matrix not being a full-rank matrix.

Reply: We thank the reviewer for highlighting this point and we agree that adding the speaker control features as separate predictors at each word onset introduced redundant predictors. We therefore removed the speaker characteristics from the analysis and report the results without these predictors. The results did not change as a result of

removing the linearly dependent predictors. We additionally removed the statement of controlling for speaker characteristics.

Page 24, Line 477.

"hlhigher" should be "higher"

<u>Reply: </u>This was fixed.

Page 24, Line 485.

delete "of"

<u>Reply: </u>This was fixed.

Page 26. Lines 531-539.

Thank you for the definition on glimpse rate. Would you please define "audibility" more clearly within this context?

<u>Reply: </u>Thank you for pointing this out. Word audibility and the glimpse rate are used analogously throughout the manuscript. We clarified this in the methods section.

l. 523:

> "Here, word audibility was operationalized as the glimpse rate, reflecting the proportion of a target word that remained locally audible despite masking by the competing speech stream."
