# Supplementary material for: Linguistic information may compensate for age-related decline in attentional filtering
Source: Commun Biol. 2026 Sep 22;9:1236. doi: 10.1038/s42003-026-10999-y (PMC13598105; doi:10.1038/s42003-026-10999-y)
Supplement: Supplementary file 2 [file 42003_2026_10999_MOESM2_ESM.pdf]

Linguistic information may compensate for age-related decline in attentional filtering

- Supplementary Material

Alice Vivien Barchet [1,2,3,*], Andrea Bruera [1], Johanna M. Rimmele [4], Jonas Obleser [5,6], and Gesa Hartwigsen [1,2]

[1] Research Group Cognition and Plasticity, Max Planck Institute for Human Cognitive and Brain Sciences, Leipzig, Germany

[2] Cognitive and Biological Psychology, Leipzig University, Leipzig, Germany

[3] International Max Planck Research School on Cognitive NeuroImaging (IMPRS CoNI), Leipzig, Germany

[4] Department of Cognitive Neuropsychology, Max Planck Institute for Empirical Aesthetics, Frankfurt, Germany

[5] Department of Psychology, University of Lübeck, Lübeck, Germany

[6] Center of Brain, Behavior, and Metabolism, University of Lübeck, Lübeck, Germany

* Corresponding Author: barchet@cbs.mpg.de

Figure S1: Pure Tone Thresholds

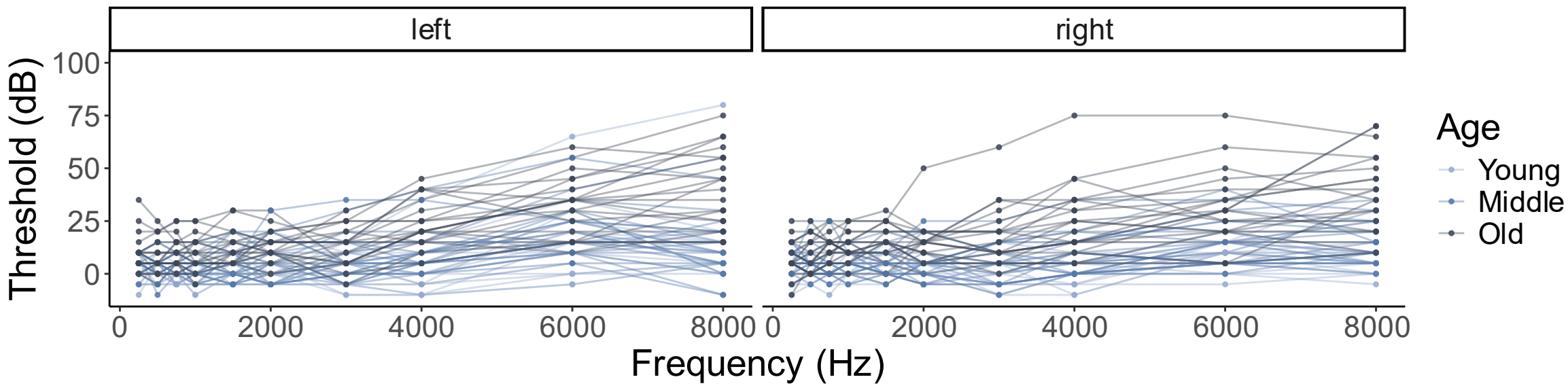

Figure S2: Correlations between Age and Confounding Variables

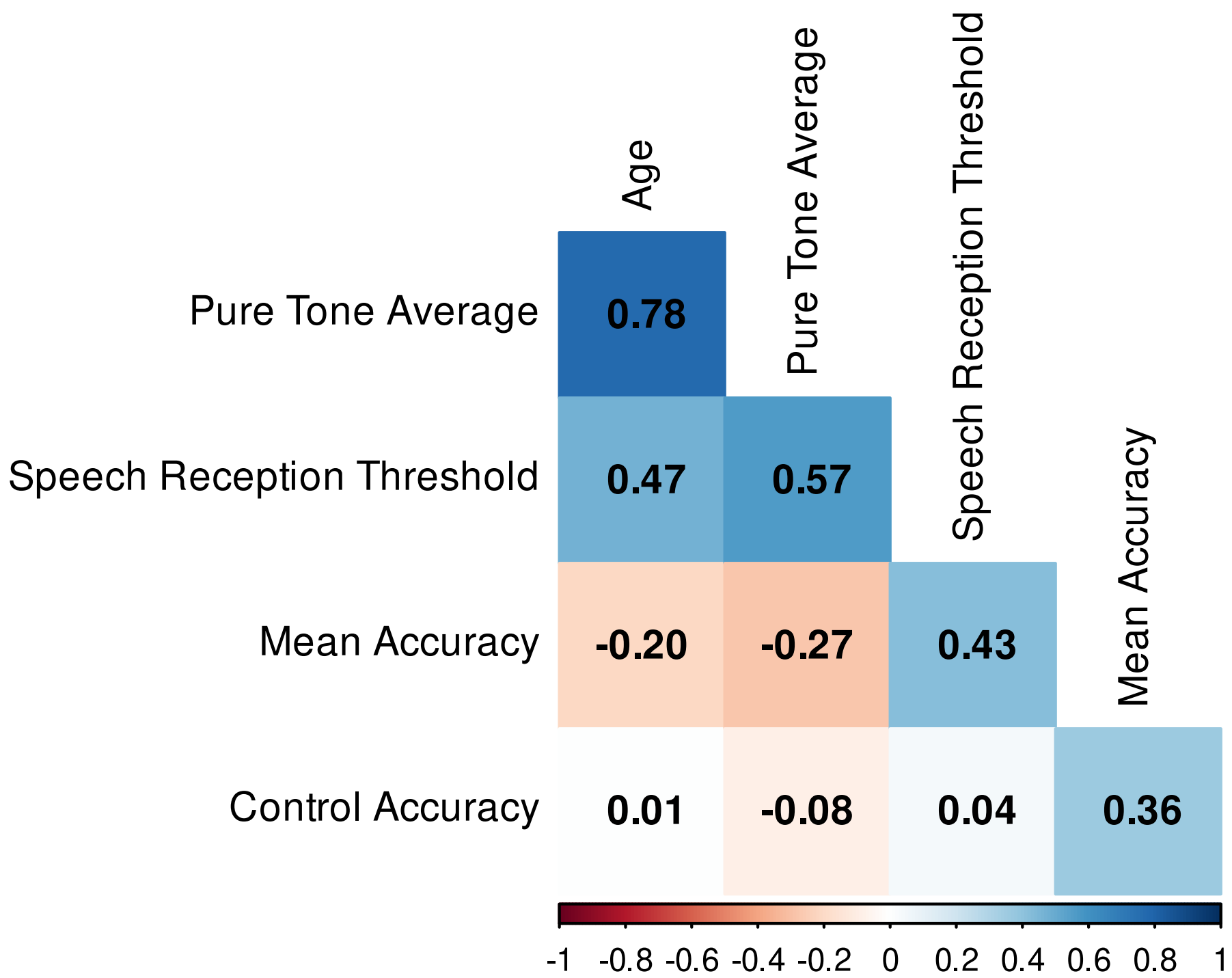

Table S1: Model Outputs for the Compensation Analysis

	Estimate	Std.Error	CI	t	p
Age	-0.62	0.09	[-0.79, -0.44]	-7.18	<0.001**
Word Target	0.3	0.09	[0.12, 0.48]	3.32	.006**
Onsets Distractor	0.16	0.08	[0, 0.32]	1.98	.114
Acoustic Distractor	0.16	0.08	[-0.01, 0.33]	1.86	.129
Word Target*Onsets Distractor	0.3	0.09	[0.12, 0.49]	3.27	.006**
Word Target*Acoustic Distractor	-0.1	0.12	[-0.34, 0.13]	-0.88	.480
Age*Word Target	0.14	0.11	[-0.07, 0.35]	1.32	.323
Age*Onsets Distractor	-0.1	0.08	[-0.26, 0.07]	-1.18	.340
Age*Acoustic Distractor	0.06	0.08	[-0.1, 0.23]	0.75	.528
Age*Word Target*Onsets Distractor	0.06	0.1	[-0.15, 0.26]	0.57	.610
Age*Word Target*Acoustic Distractor	-0.11	0.1	[-0.3, 0.08]	-1.17	.340
Control Accuracy	0.23	0.07	[0.09, 0.38]	3.17	.007**
PTA (residualized)	-0.4	0.08	[-0.56, -0.23]	-4.72	<.001**
SRT	0.31	0.03	[0.24, 0.37]	9.34	<0.001**

Model output from a linear mixed effects model on the subject level predicting subject comprehension accuracy. Statistical significance of fixed effects was assessed using two-sided t-tests with Satterthwaite approximations for degrees of freedom.
** p < .01, * p < .05 (FDR corrected)

Table S2: GPT Prompts for target sentence generation

**Prompts**
Give me 10 sentences with 5-7 words that could stem from a story.
Generate 10 everyday sentences with 5-7 words. Only use frequent words and do not use commas.
Variate the sentence structure. Do not use question marks or commas.
Generate more complex sentences with 5-7 words. Only use frequent words and do not use commas. Variate the sentence structure. Do not use question marks or commas.

Note. The same prompts were used for the distractor sentences, replacing 5-7 words with 7-10 words.

Figure S3: Neural tracking and neuro-behavioral correlations for the lower-level features resulting from a 10-fold cross-validation

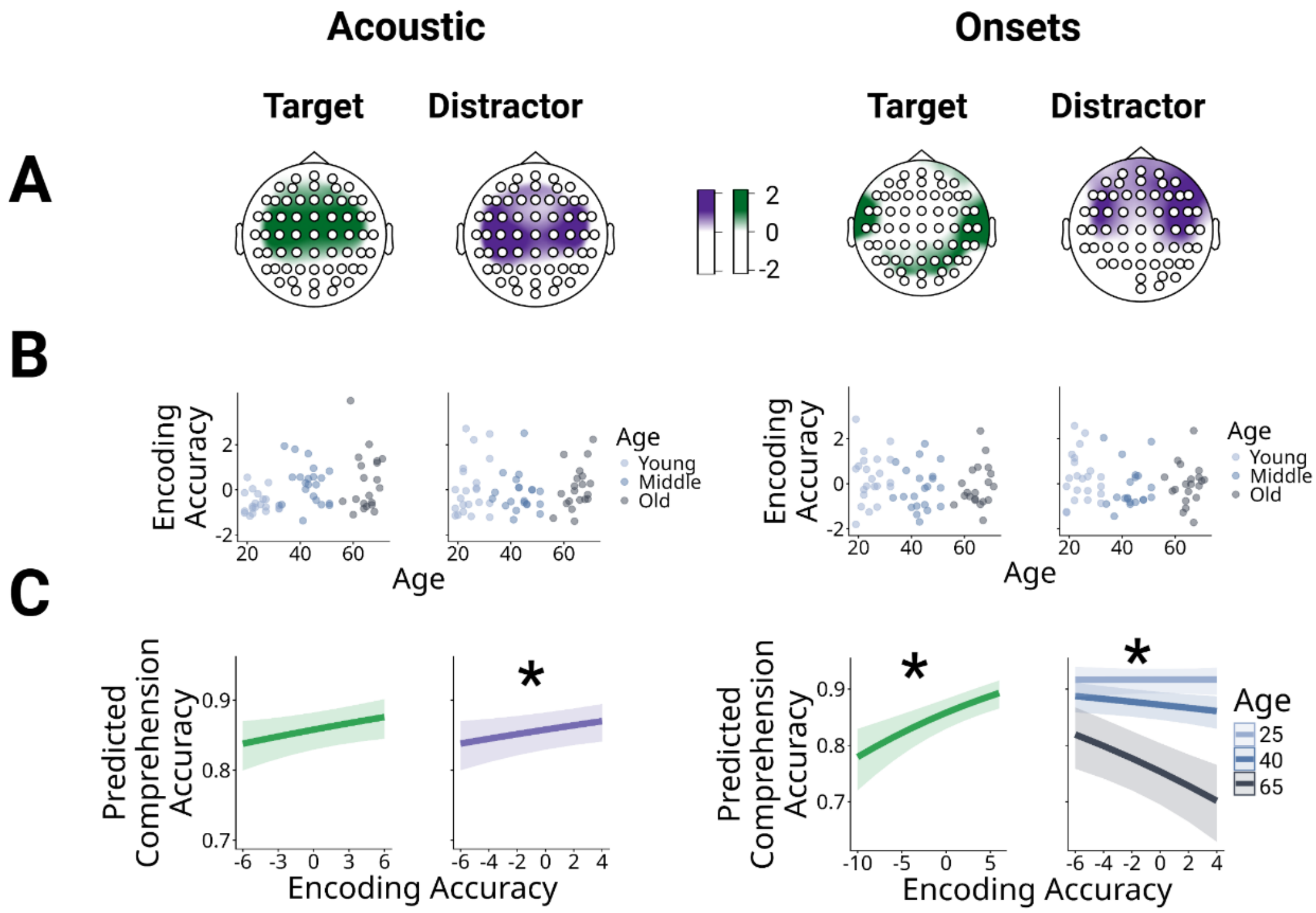

A: Topographies for the encoding accuracies. Significant electrodes in one-sided cluster-based permutation t-tests are marked by white dots. Statistical testing was performed on the participant level (N = 63 participants). B: Age effects on encoding accuracy. No significant main effects of age on encoding accuracy strength were observed. Effects were investigated using separate linear mixed effects models on the subject level predicting model fit from age. C: Neuro-behavioral correlations and age interactions. Generalized linear mixed model predictions of the effects of trial-wise neural encoding accuracy on word-by-word comprehension accuracy. Distractor acoustic features (Estimate = 0.03, SE = 0.01, p = .039*) and target onsets (Estimate = 0.05, SE = 0.01, p = <.001**) showed a positive relationship with comprehension accuracy. A significant interaction between the encoding model fits and age emerged for the distractor onsets (Estimate = -0.03, SE = 0.01, p = .024*), suggesting that older adults' comprehension performance is more negatively influenced by the neural tracking of distractor features. Statistical significance of fixed effects was assessed using two-sided Wald tests. P-values were corrected for multiple comparisons using FDR. Data are presented as the estimated marginal effects +/- 95% confidence intervals.

Figure S4: Neural tracking and neuro-behavioral correlations for the higher-level target features resulting from a 10-fold cross-validation

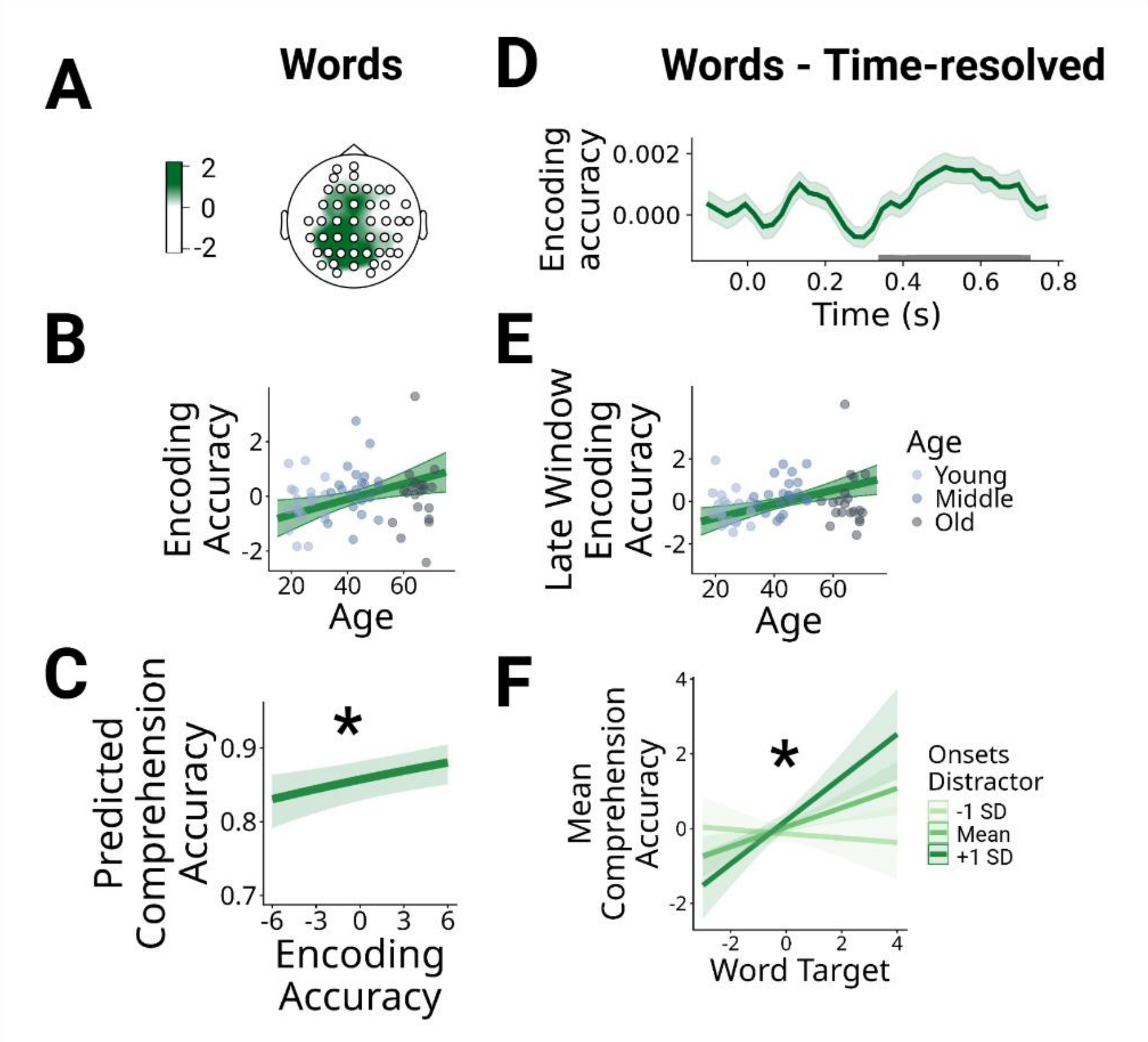

A: Topographies for the encoding accuracies. Significant electrodes in one-sided cluster-based permutation t-tests are marked by white dots. Statistical testing was performed on the participant level (N = 63 participant). B: Stronger higher-level linguistic tracking with increasing age. There was a positive correlation between age and word-level encoding accuracy (Estimate = 0.41, SE = 0.18, p = .025*). The effect was investigated using a linear mixed effects model on the subject level predicting model fit from age. C: Neuro-behavioral correlations. Generalized linear mixed model predictions of the effects of trial-wise neural encoding accuracy on word-by-word comprehension accuracy revealed a positive effect of word-level target tracking on comprehension accuracy (Estimate = 0.03, SE = 0.01, $p_{\text{FDR corrected}}$ = .012*). Statistical significance of fixed effects was assessed using two-sided Wald tests. Data are presented as the estimated marginal effects +/- 95% confidence intervals. D: Time-resolved encoding accuracy for the target stream and age effect on encoding accuracy in a late window (400 - 800 ms). Grey bars indicate significant encoding performance resulting from a one-sided cluster-based permutation one-sample t-test with threshold-free cluster enhancement. Family-wise error rate correction for multiple comparisons was achieved through the permutation-based cluster procedure. Data are presented as the mean +/- standard errors around the mean. E: Stronger late word-level tracking with increasing age. There was a positive correlation between age and word-level encoding accuracy in a late encoding window starting at 400 ms after word onset (Estimate = 0.66, SE = 0.16, p < .001**). The effect was investigated using a linear mixed effects model on the subject level predicting model fit from age. Data are presented as the estimated marginal effects +/- 95% confidence intervals. F: Word-level tracking may compensate for increased distraction. Significant interaction between the tracking of word-level target features and distractor onsets (Estimate = 0.34, SE = 0.11, t = 3.06, $p_{\text{FDR corrected}}$ = .004**) in a linear mixed effects model on the subject level predicting subject comprehension accuracy. The positive effect of word-level target tracking on comprehension accuracy was larger at high levels of distractor onsets tracking, indicating a potential compensatory function of higher-level tracking. Statistical significance of fixed effects was assessed using two-sided t-tests with Satterthwaite approximations for degrees of freedom. Data are presented as the estimated marginal effects +/- 95% confidence intervals.

Table S3: Model outputs including a predictor coding the sentence origin

	Estimate	Std. Error	CI	z	p
**Behavioral Effects**					
Age	-0.55	0.08	[-0.71, -0.39]	-6.7	<.001**
Audibility	0.41	0.01	[0.39, 0.44]	27.86	<.001**
Surprisal	-0.46	0.02	[-0.5, -0.42]	-22.25	<.001**
Audibility:Surprisal	0.17	0.02	[0.14,S 0.2]	11.08	<.001**
Word frequency	0.15	0.02	[0.1, 0.19]	6.09	<.001**
Word entropy	0.21	0.02	[0.17, 0.24]	10.95	<.001**
Audibility:Age	0	0.01	[-0.03, 0.02]	-0.38	.759
Age:Surprisal	0	0.02	[-0.03, 0.03]	0.17	.868
Age:Entropy	0.04	0.02	[0.01, 0.07]	2.8	.008**
**Lower-level TRF fits**					
Acoustic target	0.02	0.01	[-0.01, 0.04]	1.5	.168
Acoustic distractor	0.03	0.01	[0, 0.05]	2.29	.032*
Onsets target	0.05	0.01	[0.03, 0.07]	4.3	<.001**
Onsets distractor	-0.02	0.01	[-0.05, 0]	-2.12	.047*
Age:Acoustic target	0.01	0.01	[-0.01, 0.03]	0.74	.531
Age:Acoustic distractor	0	0.01	[-0.02, 0.03]	0.33	.772
Age:Onsets target	-0.02	0.01	[-0.05, 0]	-2	.060
Age:Onsets distractor	-0.03	0.01	[-0.05, -0.01]	-2.48	.020*
**Higher-level TRF fits**					
Word target	0.03	0.01	[0.01, 0.06]	2.88	.007**
Age:Word target	0.01	0.01	[-0.02, 0.03]	0.62	.594
**Control Variables**					
Pure tone threshold	-0.36	0.08	[-0.52, -0.21]	-4.68	<.001**
Target-to-distractor ratio	0.49	0.01	[0.47, 0.51]	55.87	<.001**
Trial order	0.09	0.01	[0.07, 0.12]	8.09	<.001**
Wordlength	0.23	0.02	[0.19, 0.27]	11.66	<.001**
Word order	-0.24	0.02	[-0.27, -0.2]	-13.33	<.001**
Sentence length	-0.12	0.08	[-0.28, 0.05]	-1.4	.196
Control accuracy	0.25	0.07	[0.11, 0.39]	3.43	.001**
Speech reception threshold	0.83	0.09	[0.66, 1]	9.49	<.001**
Source [GPT]	0.65	0.17	[0.32, 0.98]	3.9	<.001**

Outputs from a generalized linear mixed effects model on the word level predicting word-by-word comprehension accuracy. Statistical significance of fixed effects was assessed using two-sided Wald tests.

** p < .01, * p < .05 (FDR corrected)
