# Supplementary material for: Linguistic information may compensate for age-related decline in attentional filtering
Source: Commun Biol. 2026 Sep 22;9:1236. doi: 10.1038/s42003-026-10999-y (PMC13598105; doi:10.1038/s42003-026-10999-y)
Supplement: Supplementary file 3 [file 42003_2026_10999_MOESM3_ESM.pdf]

Description of Additional Supplementary Files

File name: Supplementary Data 1

Description: Source data is provided in supplementary data 1
