# Supplementary material for: Linguistic information may compensate for age-related decline in attentional filtering
Source: Commun Biol. 2026 Sep 22;9:1236. doi: 10.1038/s42003-026-10999-y (PMC13598105; doi:10.1038/s42003-026-10999-y)
Supplement: Supplementary file 4 [file 42003_2026_10999_MOESM5_ESM.pdf]

nature portfolio

Corresponding author(s): Alice Vivien Barchet

Last updated by author(s): May 13, 2026

Reporting Summary

Nature Portfolio wishes to improve the reproducibility of the work that we publish. This form provides structure for consistency and transparency in reporting. For further information on Nature Portfolio policies, see our Editorial Policies and the Editorial Policy Checklist.

Statistics

For all statistical analyses, confirm that the following items are present in the figure legend, table legend, main text, or Methods section.

n/a	Confirmed	
☐	☒	The exact sample size (*n*) for each experimental group/condition, given as a discrete number and unit of measurement
☐	☒	A statement on whether measurements were taken from distinct samples or whether the same sample was measured repeatedly
☐	☒	The statistical test(s) used AND whether they are one- or two-sided<br>*Only common tests should be described solely by name; describe more complex techniques in the Methods section.*
☐	☒	A description of all covariates tested
☐	☒	A description of any assumptions or corrections, such as tests of normality and adjustment for multiple comparisons
☐	☒	A full description of the statistical parameters including central tendency (e.g. means) or other basic estimates (e.g. regression coefficient) AND variation (e.g. standard deviation) or associated estimates of uncertainty (e.g. confidence intervals)
☐	☒	For null hypothesis testing, the test statistic (e.g. F, t, r) with confidence intervals, effect sizes, degrees of freedom and P value noted<br>*Give P values as exact values whenever suitable.*
☒	☐	For Bayesian analysis, information on the choice of priors and Markov chain Monte Carlo settings
☐	☒	For hierarchical and complex designs, identification of the appropriate level for tests and full reporting of outcomes
☐	☒	Estimates of effect sizes (e.g. Cohen's *d*, Pearson's *r*), indicating how they were calculated

Our web collection on statistics for biologists contains articles on many of the points above.

Software and code

Policy information about availability of computer code

Data collection	Custom code relying on the Psychophysics Toolbox Version 3 running on Matlab was used for data collection.
Data analysis	Custom code relying on MNE python and mTRFpy was used for EEG preprocessing and analysis. Statistical analysis was performed using RStudio. Linear mixed effects models were calculated using lme4 and a post-hoc simple slopes analysis was performed using the interactions package. The code used for all analyses is available at: https://github.com/vivienbarchet/compensage

For manuscripts utilizing custom algorithms or software that are central to the research but not yet described in published literature, software must be made available to editors and reviewers. We strongly encourage code deposition in a community repository (e.g. GitHub). See the Nature Portfolio guidelines for submitting code & software for further information.

Data

Policy information about availability of data

All manuscripts must include a data availability statement. This statement should provide the following information, where applicable:

- Accession codes, unique identifiers, or web links for publicly available datasets
- A description of any restrictions on data availability
- For clinical datasets or third party data, please ensure that the statement adheres to our policy

The data will be made publicly available upon publication of the manuscript.

Research involving human participants, their data, or biological material

Policy information about studies with human participants or human data. See also policy information about sex, gender (identity/presentation), and sexual orientation and race, ethnicity and racism.

Reporting on sex and gender	Our sample comprised 63 participants of whom 35 identified as female. We did not conduct any gender-related analyses or explored hypotheses concerned with sex or gender differences.
Reporting on race, ethnicity, or other socially relevant groupings	We did not obtain information on race, ethnicity or other socially relevant groupings and we did not have hypotheses, nor conducted analyses for any of these variables.
Population characteristics	All participants were German native speakers, right handed, and reported being neurologically and psychiatrically healthy. The final sample comprised 63 participants (35 women) aged 19 to 71 years (M = 43.75, SD = 17.15).
Recruitment	Participants were recruited from the participant database maintained at the Max Planck Institute for Human Cognitive and Brain Sciences, Leipzig, Germany.
Ethics oversight	The study was approved by the ethics committee of the medical faculty at Leipzig University (ethics vote: 032/24-ek).

Note that full information on the approval of the study protocol must also be provided in the manuscript.

Field-specific reporting

Please select the one below that is the best fit for your research. If you are not sure, read the appropriate sections before making your selection.

☐ Life sciences ☒ Behavioural & social sciences ☐ Ecological, evolutionary & environmental sciences

For a reference copy of the document with all sections, see nature.com/documents/nr-reporting-summary-flat.pdf

Behavioural & social sciences study design

All studies must disclose on these points even when the disclosure is negative.

Study description	We conducted an experimental quantitative study.
Research sample	All participants were German native speakers, right handed, and reported being neurologically and psychiatrically healthy. The final sample comprised 63 participants (35 women) aged 19 to 71 years (M = 43.75, SD = 17.15). Inclusion criteria included age-appropriate psychosocial functioning and hearing.
Sampling strategy	The participants were randomly selected from our in-house participant database after screening for the inclusion criteria and age. Due to the lack of comparable studies and effect sizes, we did not conduct an a priori power analysis.
Data collection	Experimental control was administered by the Psychophysics Toolbox Version 3 running on Matlab on a Windows machine. The experiment was conducted in a sound-proof, electromagnetically shielded booth. Stimuli were presented using Koss KSC75 headphones clipped to the participants' ears. Speech recordings were administered using a high quality condenser microphone (Rode NT55) at 44100 Hz.<br>EEG was recorded in a soundproof, electromagnetically shielded booth at a sampling rate of 1000 Hz using a REFA8 68-channel amplifier system (TMSi, Oldenzaal, the Netherlands), grounded to the sternum. The recording included 63 Ag/AgCl electrodes positioned according to the 10-20 layout in ANT Neuro waveguard original caps as well as two mastoid external mastoid references (A1, A2).
Timing	The data was collected from March to July 2024.
Data exclusions	4 participants were excluded after screening due to insufficient hearing performance. The exclusion criterion was defined before starting data collection.
Non-participation	No participants dropped out of the study.
Randomization	Participants were not allocated to experimental groups.

Reporting for specific materials, systems and methods

We require information from authors about some types of materials, experimental systems and methods used in many studies. Here, indicate whether each material, system or method listed is relevant to your study. If you are not sure if a list item applies to your research, read the appropriate section before selecting a response.

Materials & experimental systems

n/a	Involved in the study
☒	☐ Antibodies
☒	☐ Eukaryotic cell lines
☒	☐ Palaeontology and archaeology
☒	☐ Animals and other organisms
☒	☐ Clinical data
☒	☐ Dual use research of concern
☒	☐ Plants

Methods

n/a	Involved in the study
☒	☐ ChIP-seq
☒	☐ Flow cytometry
☒	☐ MRI-based neuroimaging

Plants

Seed stocks	*Report on the source of all seed stocks or other plant material used. If applicable, state the seed stock centre and catalogue number. If plant specimens were collected from the field, describe the collection location, date and sampling procedures.*
Novel plant genotypes	*Describe the methods by which all novel plant genotypes were produced. This includes those generated by transgenic approaches, gene editing, chemical/radiation-based mutagenesis and hybridization. For transgenic lines, describe the transformation method, the number of independent lines analyzed and the generation upon which experiments were performed. For gene-edited lines, describe the editor used, the endogenous sequence targeted for editing, the targeting guide RNA sequence (if applicable) and how the editor was applied.*
Authentication	*Describe any authentication procedures for each seed stock used or novel genotype generated. Describe any experiments used to assess the effect of a mutation and, where applicable, how potential secondary effects (e.g. second site T-DNA insertions, mosiacism, off-target gene editing) were examined.*
